# Investigating the role of tumor cell heterogeneity and angiogenesis genes in the prognosis of multiple myeloma

**DOI:** 10.3389/fimmu.2025.1610833

**Published:** 2025-06-25

**Authors:** Xue Qiao, Zhengrong Song, Li Geng, Lina Xing, Ying Wang

**Affiliations:** Department of Hematology, The Second Hospital of Hebei Medical University, Shijiazhuang, Hebei, China

**Keywords:** multiple myeloma, tumor cell heterogeneity, angiogenesis genes, prognostic model, single -cell analysis

## Abstract

**Background:**

Multiple myeloma (MM) is a common hematologic malignancy characterized by high tumor cell heterogeneity, which significantly impacts the clinical prognosis of patients. Angiogenesis and the molecular features of tumor cells play a critical role in tumor progression and drug resistance. This study aims to explore the impact of tumor cell heterogeneity and angiogenesis-related genes on the prognosis of MM.

**Methods:**

We collected transcriptomic data and single-cell RNA sequencing data from MM patients through the Xena and GEO databases. The data were processed and analyzed using bioinformatics methods, including differential gene expression analysis, single-cell clustering, CNV analysis, transcription factor analysis, screening of angiogenesis-related genes, cell communication analysis, and immune infiltration analysis.

**Results:**

Through integrative analysis of transcriptomic data and single-cell RNA sequencing data, we identified significant genomic copy number variations in the tumor cells of MM patients. Additionally, different tumor subgroups exhibited differences in angiogenic activity, gene expression, and tumor progression. Notably, high expression of the transcription factor cAMP-responsive element-binding protein 3-like 2 (CREB3L2) in the C1 subgroup was associated with the inhibition of angiogenesis and tumor cell proliferation and migration. Furthermore, the prognostic model based on angiogenesis and transcription factors demonstrated high accuracy in predicting the prognosis of MM patients.

**Conclusion:**

This study highlights the critical roles of tumor cell heterogeneity and angiogenesis-related genes in MM. By constructing a prognostic model, it provides new theoretical insights for the precise diagnosis and personalized treatment of MM.

## 1 Introduction

Multiple myeloma (MM) is a common hematological malignancy caused by the abnormal proliferation of bone marrow plasma cells. The global incidence of MM has been increasing year by year, posing a serious threat to human health. Although there have been certain advancements in current treatment methods (1–3), such as the use of proteasome inhibitors and immunomodulatory drugs, which have extended the survival period of some patients, there are still significant differences in the prognosis of MM patients. This is related to the heterogeneity of tumor cells.

Heterogeneity exists in the morphological, gene - expression, metabolic activity of tumor cells, and their differential responses to therapeutic drugs (4–6). In MM, heterogeneity leads to differences in tumor cell growth, invasion, and tolerance, resulting in varying treatment responses. Angiogenesis is one of the important mechanisms promoting tumor proliferation and metastasis and is of great significance in the development of MM. Tumor cells can obtain more nutrients and oxygen by inducing angiogenesis in a hypoxic environment, facilitating tumor cell growth and metastasis (7–9). Therefore, exploring the impact of tumor cell heterogeneity and angiogenesis genes on the prognosis of MM is a crucial step in understanding the development mechanism of MM, optimizing treatment plans, and improving patient prognosis.

Recent genomic and single-cell studies have demonstrated that MM heterogeneity is largely driven by diverse alterations in key oncogenic pathways. Activating mutations in the RAS/MAPK axis (KRAS, NRAS, BRAF) and NF-κB regulators (e.g., TNFAIP3) occur in up to 45–65% of relapsed/refractory cases and impart distinct proliferative and survival advantages to subclones (10). Integrative sequencing of 511 relapsed/refractory multiple myeloma (RRMM) patients further revealed enrichment of IL6ST mutations alongside RAS/MAPK and NF-κB aberrations, correlating with poor response to proteasome inhibitors and immunomodulatory drugs. At the single-cell level, rare drug-tolerant persister cells have been identified that upregulate ABC transporters (ABCB1, ABCG2), anti-apoptotic factors (BCL2, MCL1) and stress-responsive transcription factors such as ATF4 and XBP1, enabling survival under therapeutic pressure (11). Moreover, mutations in the proteasome subunit gene PSMB5 and in the IMiD target cereblon (CRBN) have been directly implicated in acquired resistance to bortezomib and lenalidomide, respectively (12). Beyond genetic lesions, reversible epigenetic plasticity—mediated by dysregulation of histone demethylases (e.g., KDM5 family) and chromatin remodelers—drives a slow-cycling, drug-tolerant state that underlies minimal residual disease and eventual relapse (13). Together, these findings underscore how the interplay of genetic and epigenetic programs generates functionally distinct tumor cell subpopulations that both fuel proliferation and thwart therapy in MM.

This study integrates transcriptome and single - cell RNA sequencing (scRNA - seq) data and uses multiple bioinformatics analysis methods to comprehensively and systematically analyze the characteristics of tumor cells in MM, the molecular regulatory mechanism, and its correlation with clinical prognosis (14–16). The aim is to provide a theoretical basis and potential targets for the precise diagnosis and individualized treatment of MM.

## 2 Materials and methods

### 2.1 Acquisition and processing of transcriptome data

RNA expression profiles and clinical data of multiple myeloma were collected from the UCSC Xena database (University of California, Santa Cruz, CA, USA). The Xena database is a tumor - related data repository that provides extensive data, facilitating the design of research. During data analysis, samples with a generation time of less than 10 days were excluded, and 851 samples with a generation time greater than 10 days were selected to improve the stability and reliability of the samples (17–19). The samples of MM patients included those from different stages and with different characteristics, which were highly representative.

The obtained data were converted into a measure of gene expression at the transcriptome level - transcripts per million (TPM) - which standardizes gene expression levels by converting data into the number of transcripts. This eliminates the influence of gene length and sequencing depth differences, facilitating the comparison of gene expression levels among different samples (20–22). Subsequently, the data were log2 - transformed to effectively narrow the data range, improve data normality, and facilitate subsequent statistical analysis and model construction. Part of the data was used to construct the model, and the remaining data served as the validation group to evaluate the robustness and reliability of the model, ensuring the effectiveness and stability of the research results.

### 2.2 Acquisition and processing of scRNA-seq data

The single-cell dataset was obtained from the GEO database, and GSE271107 contained 4 MM tumor samples and 5 HD (healthy control) samples, totaling 9 samples. R software (version 4.1.3), with Seurat (version 4.0.6) as the core software, was used for analysis (23–25).

During the cytoplasmic quality control stage, strict screening criteria were set: mitochondrial gene content < 20% to exclude dying or stressed cells; the unique molecular identifier (UMI) count range 200-20000 to remove barcodes with too few transcripts (ambient RNA) or potential multiplets; and gene number 200-5000 to eliminate low-complexity or doublet cells. These thresholds were chosen based on commonly used practices in cancer scRNA-seq studies and our own data distributions. A 20% mitochondrial cutoff is frequently applied to remove apoptotic cells while retaining diverse tumor subpopulations. UMI and gene count bounds follow 10x Genomics and Seurat guidelines to ensure both sensitivity for low-RNA cells and exclusion of doublets.

The Seurat package function NormalizeData was used to normalize the data, eliminating the impact of different sequencing depths of different cells. The FindVariableFeatures function was used to select 2000 highly variable genes (25–27). These genes have significant expression differences among cells and can be used to distinguish cell types. The ScaleData function was used for data transformation, and the parameters vars.to.regress = c(“S.Score”, “G2M.Score”) were set to remove the influence of the cell cycle, making the data better represent the biological state of cells.

The harmony method was applied to remove batch effects. Batch effects refer to differences between batches that can occur during experimental processes such as sample preparation and sequencing, affecting cell type identification and cell analysis results. We ran Harmony (version 1.0) on the first 20 principal components, specifying sample identity as the batch variable. We set the diversity penalty parameter theta = 2 and ridge regression penalty lambda = 0.1 (max.iter.harmony = 10), following the recommendations for moderate-heterogeneity datasets to balance integration and biological variance. After treatment with harmony, the UMAP dimensionality reduction method (UMAP) and Louvain clustering algorithm of Seurat were used to classify and cluster cells. The FindAllMarkers function was used to calculate differential genes between different clusters or cell types, with *P* < 0.05, log2 FC > 0.25, and expression proportion > 0.1 as thresholds (28–30).

### 2.3 Obtaining angiogenesis-related genes

Angiogenesis - related genes were obtained from the CancerSEA database. The information on angiogenesis-related genes in the CancerSEA database contains functional annotations of many genes related to various cancers and can be used to explore genes involved in tumor angiogenesis. These genes are important targets for subsequent data analysis, enabling the exploration of their roles in MM and their relationship with prognosis.

### 2.4 Cell annotation analysis

Cell types were annotated based on the expression of well‐established marker genes curated from the literature and public databases: epithelial cells (EPCAM, KRT8, KRT18) (31, 32); fibroblasts (DCN, LUM, FAP) (32); endothelial cells (PECAM1, VWF, CDH5) (33); T cells (CD3D, CD3E, CD8A for CD8+ T cells and CD3D, CD3E, CD4 for CD4+ T cells) (4); natural killer (NK) cells (NKG7, GNLY, KLRD1) (4); B cells (MS4A1, CD79A, CD19) (9); plasma cells (SDC1, MZB1, IGKC) (34); and myeloid cells (CD14, LYZ, FCGR3A) (35).

Using these markers, the cells in the single - cell data were classified, annotated, and UMAP plots and violin plots of cell markers were drawn. The UMAP plot intuitively reflects the distribution of cells in low - dimensional space (31–33), clearly showing the clustering characteristics of different cell types. The violin plot reflects the expression distribution of marker genes in each cell type, verifying the accuracy of cell classification and laying a foundation for further in - depth analysis of the roles of different cell types in MM.

### 2.5 Sub-group analysis of tumor cell populations

The cells corresponding to the tumor cell Cluster were separately extracted, and topMarker was used for further cell annotation analysis. topMarker refers to genes that are highly expressed or specifically expressed in tumor cell sub - populations. Through the analysis of TopMarker, a more in - depth study of the characteristics and differences of tumor cell sub - populations can be carried out, revealing the biological characteristics of different tumor cell sub - groups and providing more detailed information for the study of tumor cell heterogeneity.

### 2.6 Single - cell CNV analysis

The InferCNV software was used to perform CNV (Copy Number Variation) analysis on each cell sub - population. Normal plasma cells were used as a reference, and the genome of normal plasma cells served as a stable reference standard for comparing the genomic changes of tumor cell sub - populations.

This analysis can accurately identify malignant cells with abnormal genomic copy numbers and evaluate the CNVscore of each cell sub - population. The CNVscore quantifies the degree of genomic copy number variation in cells (34–36). The magnitude of its value represents the instability of the cell genome, providing guidance for understanding the genomic variation characteristics of tumor cells and evaluating the malignancy of tumor cells.

### 2.7 Single - cell pseudotime analysis

The monocle2 software (v2.18.0; Seattle, USA) was used to perform pseudotime analysis on tumor cell sub - populations to simulate the cell differentiation pathway. In the pseudotime analysis, the dimensionality reduction algorithm was set as discriminative dimensionality reduction via trees (DDRTree). The DDRTree algorithm can effectively capture the high - dimensional non - linear structure of cells and map it to a low - dimensional space, more appropriately expressing the relationships and differentiation order among cells. Other parameters were set by default to ensure the standardization and repeatability of the analysis process.

Pseudotime analysis can present the kinetic changes of tumor cells from the initial state to various differentiated states, determine the positions and rules of each cell sub - population in the differentiation trajectory, and understand the differentiation mechanism and evolution rules of tumor cells, providing a reference for tumor development.

### 2.8 Transcription factor analysis of MM cells

The SCENIC software (v1.2.0; Seattle, USA) was used to analyze the transcription factors of tumor cell sub - populations. The SCENIC software integrates transcription factor binding site data and gene expression data and can directly infer the transcription factor control network. The default parameters in the motif databases of RcisTarget (v1.2.0; Seattle, USA) and GRNBoost were used for SCENIC analysis.

The RcisTarget software package was used to identify significantly over - expressed transcription factor binding motifs in the gene list. These binding motifs are important components for transcription factors to exert regulatory functions. By identifying binding motifs, transcription factors with potential regulatory activity in MM tumor cell sub - populations can be determined. The activity of each regulator group for each cell type was scored using the AUCell software (v1.12.0; Seattle, USA) package to quantify the level of transcription factor activity, providing a numerical quantification index for further exploring the regulatory mechanism of transcription factors in MM cells.

Transcription factor regulatory network inference was conducted using SCENIC (v1.2.0), integrating single‐cell expression data with cis‐regulatory motif information. To tailor the analysis for MM data, we optimized key SCENIC parameters rather than relying solely on defaults. First, the GRNBoost2 algorithm (implemented in the pySCENIC pipeline) was run with 1 000 trees (n_estimators = 1000) and a minimum gene–gene co‐expression correlation threshold of 0.001, which we determined by inspecting the distribution of pairwise gene correlations in our MM dataset to balance sensitivity for weak but biologically relevant co‐expression links against noise. Next, putative regulons were refined using RcisTarget (v1.2.0) with the hg38 motif rankings database (mm9 version 10 kb upstream, motif ranking file “hg38‐500bp.owl”); we retained only those transcription factor (TF)–target motif enrichments meeting an FDR < 0.05 and an enrichment score (NES) > 3. Finally, regulator activity was quantified per cell with AUCell (v1.12.0; USA) using an AUC threshold of 0.05 (i.e., a gene set was considered “active” in a cell if its AUC score exceeded the 95th percentile of background AUC values for shuffled gene sets), which we selected by plotting the bimodal distribution of AUC scores in our MM single‐cell data and choosing the inflection point between low‐ and high‐activity modes.

To validate the inferred regulons, we cross‐referenced our top TF–target pairs against two independent resources: DoRothEA v2 regulon database (confidence levels A–C) and JASPAR 2022 (core vertebrates collection). We found that > 80% of high‐confidence TF–target interactions identified by SCENIC overlapped with entries in DoRothEA or had corroborating evidence in JASPAR (e.g., matching PWM hits within ± 10 kb of the transcription start site), thereby confirming the biological relevance of our MM‐specific regulons. In addition, we compared the activity patterns of canonical MM‐related TFs (e.g., XBP1, IRF4) against published single‐cell studies in plasma‐cell malignancies (37), observing consistent enrichment in tumor subpopulations known to depend on these regulators. All parameter settings and validation metrics are provided here to enhance reproducibility and confidence in our SCENIC‐based regulatory network inferences.

### 2.9 Cell - to - cell communication analysis

The CellChat package (v1.4.0; Jinmiao Chen Lab, Shanghai, China) was used to analyze the communication status between cells. First, the normalized gene expression matrix was imported through the CellChat function to obtain a cellchat object, providing raw data for the next - step analysis.

Then, the functions identifyOverExpressedGenes, identifyOverExpressedInteraction, and ProjectData were used for data pre - processing. The function identifyOverExpressedGenes is used to find over - expressed genes, the function identifyOverExpressedInteraction is used to identify over - expressed cell - to - cell interactions, and the function ProjectData is used to project the data for the next - step cell communication analysis.

Subsequently, potential ligand - receptor interactions were identified by calculating communProb, filterCommunication, and communProbPathway functions. The communProb function calculates the cell - to - cell communication probability, filterCommunication filters the communication probability to remove low - confidence interactions, and the communProbPathway function further determines the communication pathway.

Finally, the aggregateNet function was used to visualize the generated cell communication network, allowing for direct observation of the communication relationships between different cell types. This provides evidence for the study of information transfer and collaborative regulation between cells and is conducive to further clarifying the mutual regulatory effects among cells in the MM tumor microenvironment.

### 2.10 Calculation of angiogenesis signature score

Using angiogenesis - related genes, the single-sample gene set enrichment analysis (ssGSEA) algorithm of the GSVA package (v1.40.1; Seattle, USA) was used to calculate the functional score of single - cell data. The ssGSEA (single - sample Gene Set Enrichment Analysis) algorithm can calculate the gene - set enrichment score of a single sample based on the expression of a gene set in that sample, quantifying the activity of angiogenesis at the single - cell level.

The angiogenesis signature score can be used to judge the overall expression of angiogenesis - related genes in each single cell, facilitating the quantitative study of the role of angiogenesis at the single - cell level in MM and helping to understand the differences in angiogenesis activity among cells and its association with tumor cell heterogeneity.

### 2.11 Immune infiltration analysis

The ESTIMATE (v1.0.13; New York, USA), CIBERSORT (v1.06; Stanford University, USA), and xCell (v1.1.0; Stanford University, USA) algorithms were called through the IOBR package (v0.99.9; Beijing, China) to quantitatively evaluate the immune infiltration level of each patient in the risk group. The ESTIMATE algorithm evaluates the degree of tumor immune infiltration based on the content of immune cells and stromal cells in the tumor microenvironment. CIBERSORT can accurately infer the content of 22 types of immune cells in tumor tissues using gene expression profiles. xCell provides more comprehensive annotation information of immune cells and stromal cells for quantitative analysis of multiple cell types.

By comprehensively applying these three algorithms, a comprehensive evaluation of the immune infiltration in the tumor tissues of MM patients was carried out from multiple dimensions, including the types, numbers, and proportions of immune cells, and the relationship between the immune system and tumors was explored in depth, providing a basis for guiding the application of immunotherapy in MM.

### 2.12 Differential gene analysis and enrichment analysis

The limma software was used to perform differential gene analysis on the high - and low - Risk groups. The limma software is widely used for gene expression data analysis and has powerful statistical analysis functions. Strict differential screening conditions were set in the limma program software: |logFC| > 1 and padj < 0.05. Here, |logFC| (log Fold Change) represents the multiple of gene expression changes between the two groups, and padj (adjusted *P* - value) is the p - value corrected by multiple tests, used to control the false - positive rate. Genes that meet these two conditions are considered to have differential expression between the high - and low - Risk groups.

The clusterProfiler package (v4.2.2; Yu Lab, Guangzhou, China), Kyoto Encyclopedia of Genes and Genomes (KEGG), and Gene Ontology (GO) libraries were used for enrichment analysis. The KEGG database contains rich biological pathway information, and the GO database contains gene function annotations, cell components, and biological process information. When the *P* - value after BH (Benjamini - Hochberg) correction is less than 0.05, the gene is determined to be enriched in that function. Finally, the ggplot2 package was used for visualization, presenting the results of the enrichment analysis in an intuitive chart form, facilitating the intuitive observation and analysis of the enrichment of differential genes in biological functions and signaling pathways and discovering potential biological differences between the high - and low - risk groups.

### 2.13 Comparison of genomic variation landscapes between two groups

The R package “maftools” was used to manipulate mutation data for mutation burden difference analysis between the two groups. The “maftools” package can read and write mutation data files (such as MAF - format files) and has multiple analysis and visualization functions. Using this package, the mutation frequency, mutation type distribution, and genomic mutations of tumor cells in the two groups of samples can be calculated.

At the same time, the “maftools” package was used to draw a mutation waterfall plot. The waterfall plot can directly reflect the mutated genes, mutation types, mutation sites in each sample, and the distribution of mutations in the sample, clearly showing the characteristics and differences of genomic variations. It is a visualization tool for observing and intuitively indicating the differences in genomic variation landscapes between the two groups and can be used to search for important mutated genes affecting the prognosis of MM.

### 2.14 Establishment of a LASSO - Cox prognostic model based on transcription factors and angiogenesis genes of C1 tumor cells

A total of 75 genes, including TARGET genes (importance > 10) related to the specific transcription factor cAMP-responsive element-binding protein 3-like 2 (CREB3L2) of C1 tumor cells and angiogenesis genes, were extracted. First, a univariate Cox analysis was performed. The Cox proportional - hazards model is a commonly used survival analysis method used to evaluate the relationship between a single gene and the survival outcome. Through univariate Cox analysis, genes related to survival were selected, and candidate genes with prognostic value were initially selected.

Then, the Least Absolute Shrinkage and Selection Operator (LASSO) + Cox algorithm was used to construct a prognostic model with the glmnet (v4.1-3; Friedman Lab, Stanford University, USA) software package. The LASSO algorithm can compress and select regression coefficients during model construction, avoiding overfitting and selecting the gene combination with the most predictive value for prognosis. After final modeling, the risk score of patients can be calculated based on their gene expression data to predict their survival status.

Finally, the timeROC (v0.4; Blagus Lab, University of Ljubljana, Slovenia) package was used to estimate the AUC (Area Under The Curve) values at 1, 3, and 5 years. The AUC value is used to evaluate the prediction accuracy of the model, with a value range of 0 - 1. The closer the AUC value is to 1, the higher the prediction accuracy of the model. By comparing the AUC values at different time points, the prediction accuracy of the model at different follow - up time points can be fully evaluated, which is helpful for the selection of the model in clinical applications.

### 2.15 Statistical analysis

All data analysis and statistical mapping were performed using R4.1.3 software. The Pearson correlation coefficient was used to test the linear relationship between two continuous variables. The value of the Pearson correlation coefficient ranges from - 1 to 1, and the closer it is to 1, the stronger the correlation. The chi - square test was used to compare categorical variables, which is used to determine whether there is an association between two or more groups of categorical variables. The Wilcoxon rank - sum test or T - test was used to compare continuous variables. The Wilcoxon rank - sum test is applicable to continuous variables that do not follow a normal distribution, and the T - test is applicable to continuous variables that follow a normal distribution. The appropriate test method was selected according to the characteristics of the research data to ensure the accuracy of the statistical results. A *P* value < 0.05 was considered statistically significant. Data are presented as mean ± standard deviation (SD) unless stated otherwise.

The survminer (v0.4.9; Kassambara Lab, University of Auckland, New Zealand) package was used to determine the optimal cut - off value. In survival analysis, the optimal cut - off value is used to divide continuous variables (such as risk scores) into different categories to distinguish high - risk and low - risk groups. Both Cox regression and Kaplan - Meier analysis were performed using the survival package. Cox regression was used to establish a multi - factor survival model to examine the comprehensive impact of multiple factors on the survival outcome. Kaplan - Meier analysis was used to create survival curves, intuitively observing the survival status of patients in different groups and comparing whether there are differences in survival rates between groups, providing a comprehensive statistical analysis for studying the prognosis of MM patients.

## 3 Results

### 3.1 Single - cell expression atlas of MM

**Figure 1:**
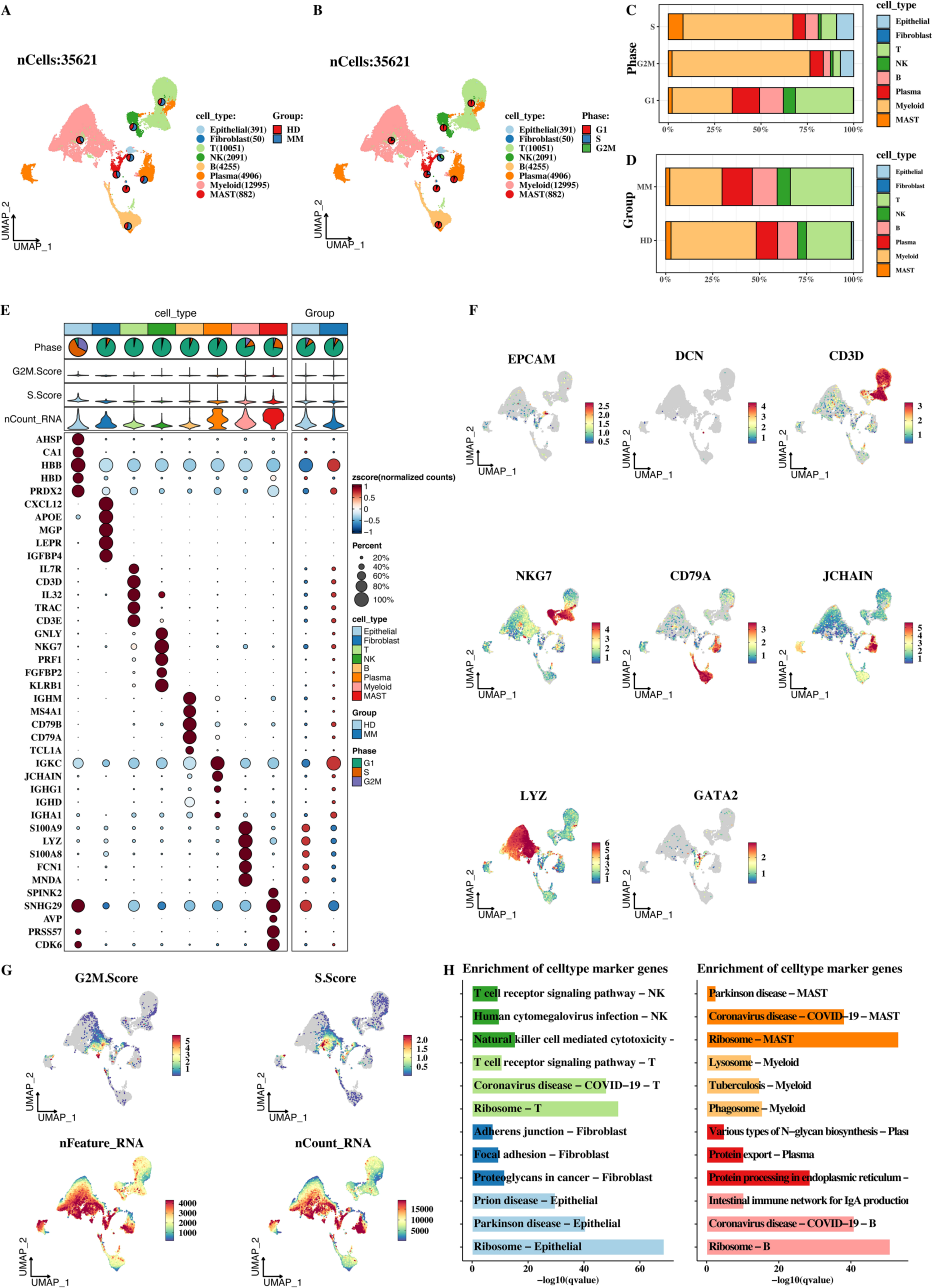
Single-cell expression atlas of MM. **(A)** UMAP analysis of single cells. **(B)** Proportional distribution of different cell types in MM samples. **(C)** Distribution of different cell types across cell cycle stages. **(D)** Proportional differences in cell types between MM and healthy donor samples. **(E)** Bubble plot of marker gene expression in different cell types, where bubble size and color intensity reflect gene expression levels. **(F)** UMAP spatial distribution of marker genes in different cell types. **(G)** Distribution of G2M.score, S.score, nFeatureRNA, and nCountRNA in different cell types. **(H)** KEGG enrichment analysis of marker genes for each cell type.

After a series of analysis processes such as strict quality control and fine - grained dimensionality reduction of single - cell data, a total of 35621 high - quality cells were finally obtained. Based on classical cell - classification markers, these cells were successfully divided into 8 major categories: epithelial cells, fibroblasts, endothelial cells, T cells, NK cells, B cells, plasma cells, and myeloid cells (**Figures 1A, B**). This classification result laid the foundation for subsequent studies on the roles of different cell types in MM. In addition, the distribution proportions of cell types in the cell cycle and sample types were shown (**Figures 1C, D**). In terms of cell types, it can be seen from the cell cycle that different cell types have different distributions in different stages of the cell cycle, that is, their proliferative activities and metabolic states are different. From the perspective of sample types, the proportions of each cell category in MM tumor samples and HD samples also vary, indicating that the tumor microenvironment can affect cell type distribution.

The bubble plot (**Figure 1E**) and UMAP plot (**Figure 1F**) clearly reflect the marker expression of different cell types. In the bubble plot, the larger and darker the bubble, the higher the gene expression level and the greater the proportion of cells expressing the gene. It can be seen that the expression intensity and prevalence of marker genes in different cell types vary. The UMAP plot reflects the expression of marker genes in different cells from the spatial distribution, which can verify the correctness of cell classification and ensure the accuracy of cell type division in the analysis. In addition, the distributions of G2M.score, S.score, nFeatureRNA, and nCountRNA in each cell were shown (**Figure 1G**). G2M.score and S.score reflect the stages of the cell cycle. The distribution differences of different cell types can help us understand the proliferative states of different cell types. nFeatureRNA represents the number of genes detected in each cell, and nCountRNA represents the number of UMI in each cell. Their distributions reflect the richness of gene expression in cells and the depth of sequencing. At the same time, the KEGG enrichment analysis of the markers of each cell type was presented (**Figure 1H**). Through enrichment analysis, we found that the marker genes of different cell types are enriched in different biological processes and signaling pathways.

### 3.2 Analysis of CNV

**Supplementary Figure 1** shows the heat map of the results of analyzing plasma cells using the inferCNV software (v1.9.1; MA, USA). The abscissa represents the genomic region, and the ordinate is not marked but should be related to sample classification. “HD” represents healthy control samples, and “MM” represents multiple myeloma samples. The color depth in the figure represents the change in gene expression level. The more the color deviates from the middle color (such as the deeper the red or blue), the more significant the change in gene expression level. It can be seen that there are differences in the gene expression corresponding to the genomic copy number between MM samples and HD samples, intuitively indicating that tumor cells (plasma cells in MM samples) have very significant CNV changes compared with normal plasma cells (HD samples). This change provides important clues for subsequent studies on the malignant characteristics and related mechanisms of tumor cells.

**Supplementary Figure 2** shows the analysis results of plasma cell subsets. **Supplementary Figures 2A, B** are UMAP dimensionality - reduction plots, involving a total of 4906 cells. In **Supplementary Figure 2A**, cells are marked with different colors according to different “seurat_cluster” (clusters 0 - 8), and it can be seen that the cells are divided into multiple subsets. In **Supplementary Figure 2B**, cells are marked according to sample groups. Light blue represents healthy control samples (HD, a total of 1986 cells), and dark blue represents multiple myeloma samples (MM, a total of 2911 cells). It can be clearly seen that the cells from MM and HD samples have obvious clustering boundaries on the UMAP plot.

**Supplementary Figures 2C, D** are violin plots used to show the relationship between CNVscore (copy number variation score) and gene expression level. **Supplementary Figure 2C** shows the distribution of CNVscore in different cell subsets according to “seurat_cluster”, and the distribution of CNVscore in each subset is clear at a glance. **Supplementary Figure 2D** shows the distribution of CNVscore in MM samples (dark blue) and HD samples (light blue) according to sample groups. It can be seen that there are differences in the CNVscore distribution between MM samples and HD samples. Combining the UMAP plot and CNVscore analysis, the left - hand cluster shows different CNVscore characteristics from other subsets and HD samples. Therefore, it can be judged that the left - hand cluster is tumor/malignant cells, providing intuitive data support for further in - depth study of the malignant characteristics and mechanisms of plasma cells.

### 3.3 Tumor subtype-specific characteristics and heterogeneity of malignant cell populations

**Figure 2:**
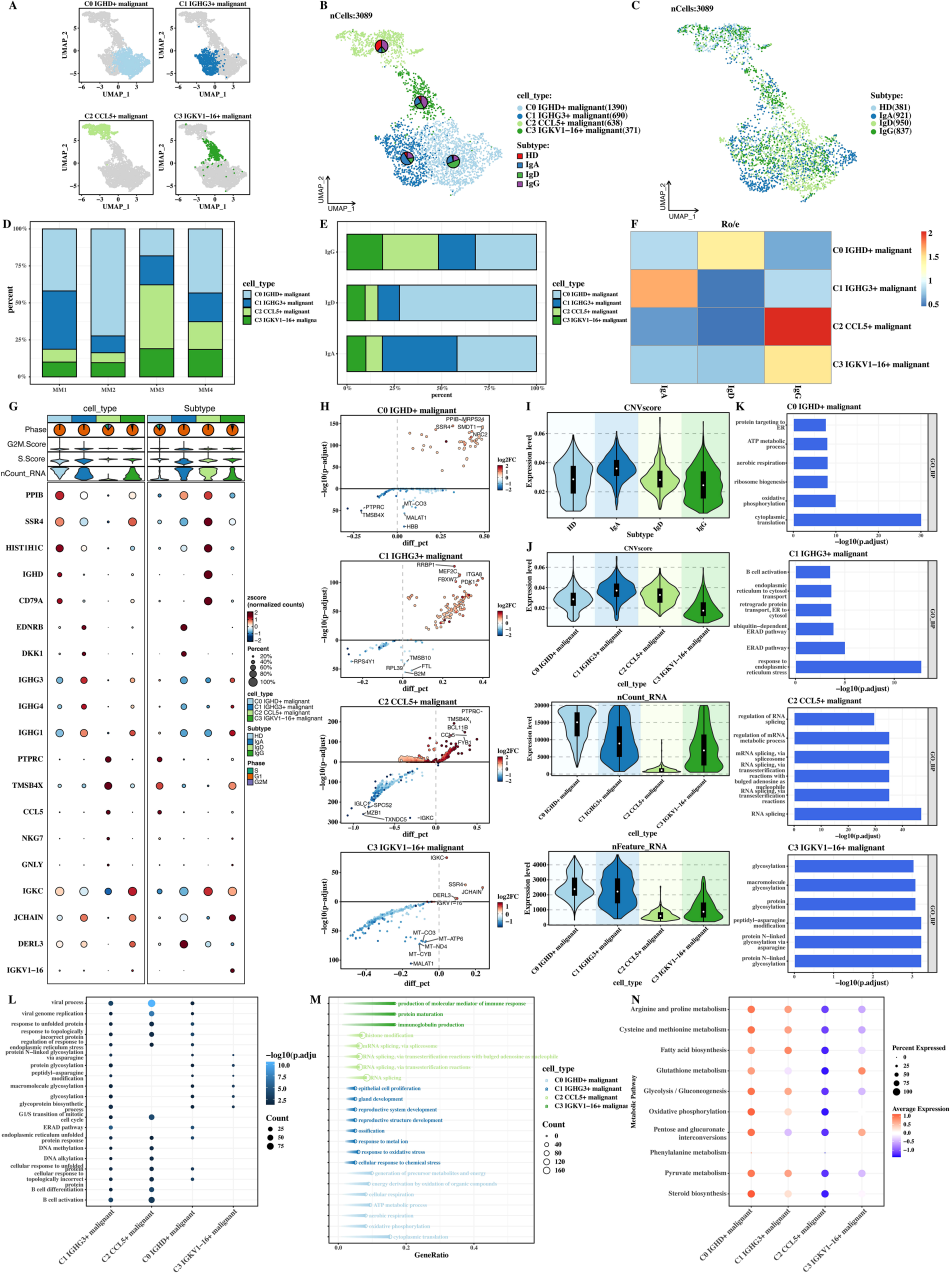
Tumor subtype-specific characteristics and heterogeneity of malignant cell populations. **(A)** UMAP plot of four malignant cell populations (C0, C1, C2, C3). **(B)** UMAP plot of tumor subtypes (HD, IgA, IgD, IgG). **(C)** Proportional distribution of malignant cell populations across tumor subtypes. **(D)** Malignant cell population distribution within IgA, IgD, and IgG subtypes. **(E)** Ro-Ro E-value heatmap showing preferential distribution of cell populations across subtypes. Each cell shows the ro-ro e-value, defined as –log10 of the reciprocal BLAST E-value between receptor pairs. Higher values (red) indicate stronger reciprocal best-hit similarity; lower values (blue) indicate weaker or non-significant alignments. **(F)** Marker gene expression heatmap of malignant cell populations. **(G)** Volcano plots of differential gene expression across cell populations. **(H)** CNVscore distribution across cell populations. **(I-K)** GOBP enrichment analysis of marker genes. **(L)** Bubble plot of common enrichments in cell metabolism and signaling pathways. **(M)** Arrow plot of unique biological processes in each malignant cell population. **(N)** ssGSEA scores for metabolic pathways.

The malignant cells were isolated separately, subjected to dimensionality - reduction clustering, and the four malignant cell populations obtained by clustering were named according to topMarker. According to tumor subtypes, the cells were divided into Immunoglobulin A (IgA), Immunoglobulin G (IgG), and Immunoglobulin D (IgD), and their UMAP plots and composition results were shown respectively (**Figures 2A–E**). The distribution characteristics and differences of different subtypes of malignant cell populations in the low - dimensional space can be seen from the UMAP plot. The compositions of each subtype cell population are also different, reflecting the heterogeneity at the tumor cell subtype level.

The heat map of ro-ro e - value (**Figure 2F**) shows that there are obvious differences in the tumor subtype attribution of different malignant cell populations. The ro-ro e - value heat map can reflect the preferential distribution of cell populations in different tumor subtypes, providing a quantitative basis for further clarifying the relationship between malignant cell populations and tumor subtypes. Then, the marker expressions of different cell populations were shown (**Figure 2G**). The marker gene expressions of different cell populations are different, which can not only distinguish different cell populations but also imply differences in their biological functions.

The differential genes of each cell population were shown through volcano plots (**Figure 2H**), intuitively showing the gene expression changes among different cell populations. In the figure, red dots represent up - regulated genes, blue dots represent down - regulated genes, and the larger and darker the dots, the greater the gene expression difference. Through the comparison of CNVscore and other indicators of each cell population in different subtypes (**Figures 2I–K**), it was obtained that C1 has the largest CNVscore, indicating that the genomic variation degree of the C1 cell population is the highest and may have greater malignant potential. The GOBP enrichment results of the markers of each cell population (**Figures 2J–K**) can show the differential enrichment results of the marker genes of different cell populations in biological processes (GOBP).

The common enrichment results among cell populations were presented using a bubble plot (**Figure 2L**), and it can be seen that many cell populations have common enrichments in some cell metabolism and signal transduction pathways, which may be the common mechanism for tumor cells to survive or maintain their basic survival functions. The unique enrichment results of each cell population were presented using an arrow plot (**Figure 2M**), visually showing the unique biological processes and functions of each cell population and emphasizing the differences among cell populations. Finally, the ssGSEA scores of metabolic pathways in KEGG were calculated and their distributions in each cell population were presented (**Figure 2N**). It can be seen that there are differences in metabolic activities and the utilization of metabolic pathways among different cell populations.

### 3.4 Trajectory analysis of tumor cells

**Figure 3:**
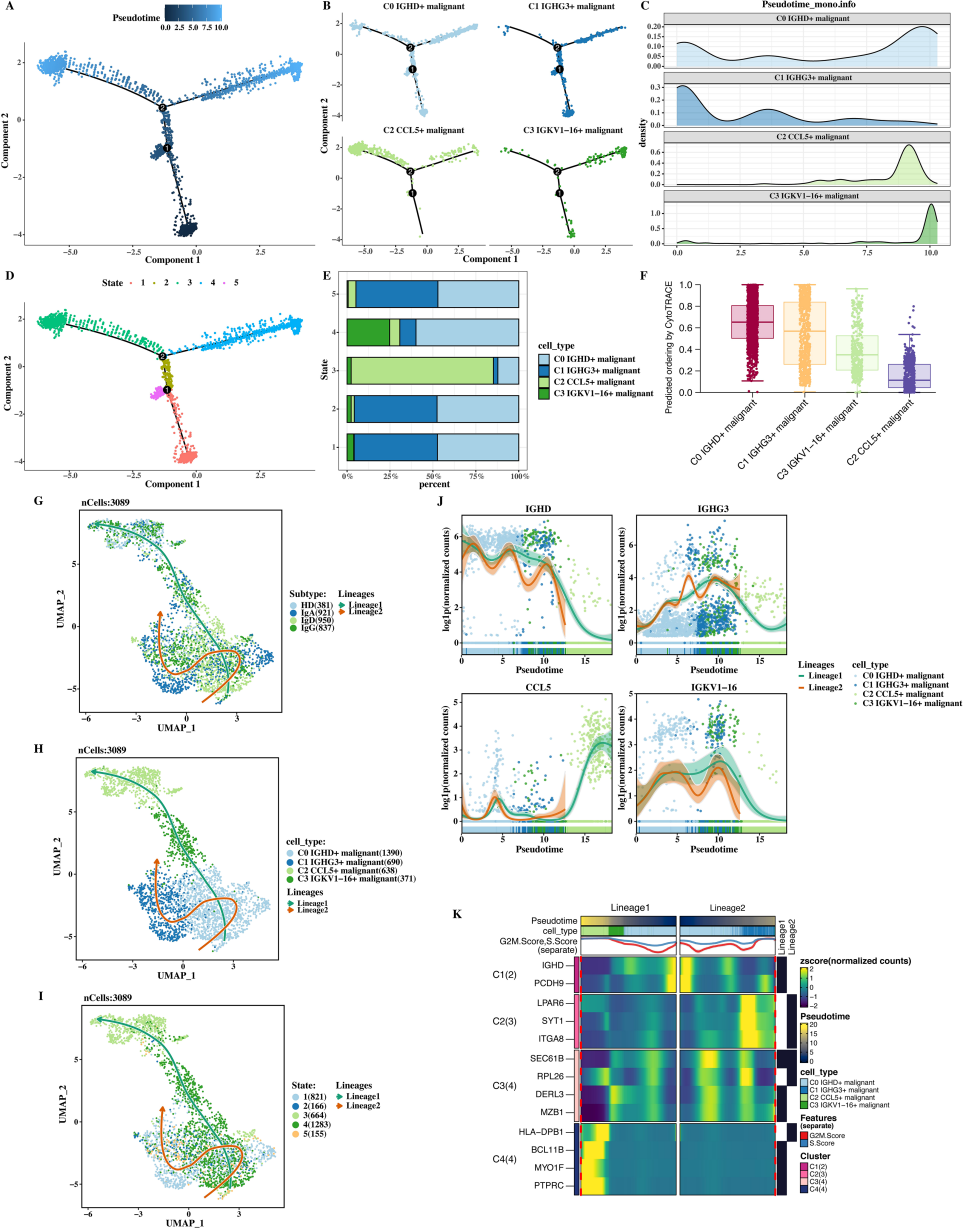
Trajectory analysis of tumor cells. **(A)** Trajectory plot showing differentiation from C1 (early stage) to C2 and C3 (later stages). **(B)** Expanded trajectory plot showing tumor cell progression from C0 to C3. **(C)** Pseudotime distribution of tumor cells along the differentiation path. **(D)** Cell composition analysis across states (1–5), highlighting differences in tumor cell populations. **(E)** CytoTRACE analysis confirming C1 as the early differentiation stage. **(F)** Slingshot analysis showing two distinct tumor cell differentiation trajectories. **(G)** UMAP plot of tumor cells along two differentiation lineages. **(H)** UMAP plot showing tumor cells in different differentiation states (1–5). **(I)** TopMarker gene expression changes over pseudotime. **(J)** Gene expression of IGHD, IGHG3, CCL5, and IGKV1−16 across pseudotime. **(K)** Heatmap of genes correlated with pseudotime.

The trajectory analysis of tumor cells was performed using monocle2, and a clear trajectory plot was obtained (**Figures 3A–C**). The results showed that C1 was in the early stage, and C2 and C3 were in the late stage. All cells were divided into 5 States. From the trajectory plot, it can be directly seen that the tumor cells differentiate from the initial form to endpoints with different degrees of differentiation and development over time, as well as the positions of tumor cells in the cell population and the differentiation trajectory. The cell compositions of each State were shown (**Figures 3D, E**), and it can be seen that there are differences in the subtype and percentage of cell populations among different States, indicating the heterogeneity of the tumor cell differentiation process.

The CytoTRACE software (v0.3.3; Boston, USA) was used to accurately measure the differentiation stages of each cell population (**Figure 3F**), confirming the accuracy of C1 being in the starting/early stage. The CytoTRACE software provides supporting evidence for determining the starting position of cells in the differentiation trajectory by measuring the differentiation potential of cells. The slingshot software (v2.2.0; Cambridge, UK) was used to analyze the trajectory, and two clear trajectories were obtained (**Figures 3G–I**). These two trajectories may represent different differentiation directions or development paths of tumor cells, providing new clues for studying the differentiation mechanism of tumor cells from a new perspective.

Finally, the expression of topMarker of each cell population over pseudotime was shown (**Figure 3J**). The expression of topMarker of different cell populations changes dynamically with the progress of pseudotime, indicating the change of gene expression regulation during cell differentiation. Genes correlated with pseudotime were calculated and their expression was shown in a heat map (**Figure 3K**). The color depth in the heat map represents the level of gene expression. From the heat map, the change trends of different genes at different time points can be clearly and intuitively seen, which is helpful for screening key regulatory genes and molecular mechanisms during the differentiation process of tumor cells.

### 3.5 Cell communication analysis

**Figure 4:**
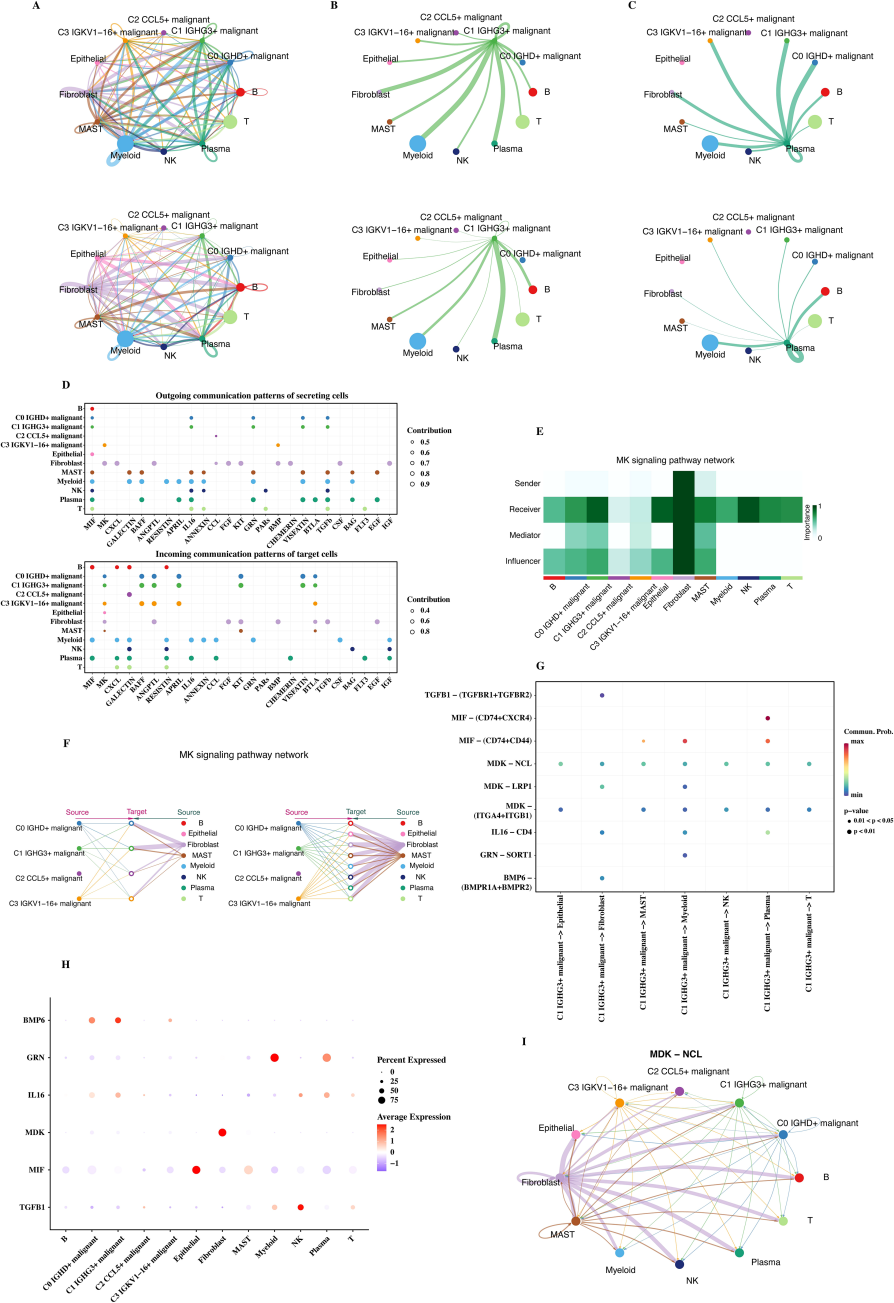
Cell communication analysis. **(A)** Communication network diagrams for C1, C2, and C3 malignant cells. **(B)** Communication patterns between malignant cells and immune/non-immune cells. **(C)** Comparison of communication networks across different malignant cell subtypes. **(D)** Bubble plots showing the incoming and outgoing signals of each cell group in various pathways. **(E)** Communication heatmap of the PTN pathway. **(F)** MK signaling pathway network depicting communication between cell types across malignant groups. **(G)** Bubble plot of communication from C1 malignant cells to other cells. **(H)** Ligand gene expression in C1 cells, shown through single-cell data. **(I)** MDK-NCL ligand-receptor network.

CellChat was used to analyze the communication status of each cell subtype, and the communication network diagrams of each cell subtype were shown (**Figures 4A–C**). In the communication network diagram, the thickness of the lines represents the communication intensity between cells. It can be seen from the figure that the communication pattern of C1 malignant cells (with high CNVscore and in the initial stage of trajectory analysis) is different from that of other cells. C1 malignant cells have strong communication connections with some cells, indicating that they can interact with other cells in the tumor microenvironment and affect tumor progression. Normal plasma cells have a special communication pattern with other cells, including immune cells, and may play a role in immune regulation.

Bubble plots of incoming and outgoing signals of each cell group in each pathway were shown (**Figure 4D**). The size and color of the bubbles represent the intensity of the communication signal. From the bubble plot, the communication strengths of different cell groups in different signal pathways can be directly and intuitively analyzed.

The PTN pathway (with more communication between C1 malignant and normal plasma cells) was selected, and its communication heat map (**Figure 4E**) and the communication situation diagram of each cell (**Figure 4F**) were shown. The darker the color in the communication heat map, the stronger the ligand - receptor interaction. It can be seen from the figure that there is a specific communication pattern between C1 malignant cells and normal plasma cells in the PTN pathway. The communication situation diagram of each cell directly reflects the specific communication situation between different cells on this pathway.

A bubble plot of the communication from C1 malignant cells to other cells was given (**Figure 4G**). It was observed that C1 malignant cells communicate with many other types of cells, and the communication intensities on different types of cells are different. The ligand genes appearing in the above figures were observed through a bubble plot for their expression in single - cell data (**Figure 4H**), and it was found that MDK - NCL was involved in the communication situation in **Figure 4G**. Finally, the cell communication network diagram of the MDK - NCL ligand - receptor was given (**Figure 4I**). The communication network diagram of the MDK - NCL ligand - receptor provides the specific communication pattern and potential regulatory mechanism between cells, providing clues for understanding the information transfer between tumor cells and other cells.

### 3.6 Transcription factor analysis

**Figure 5:**
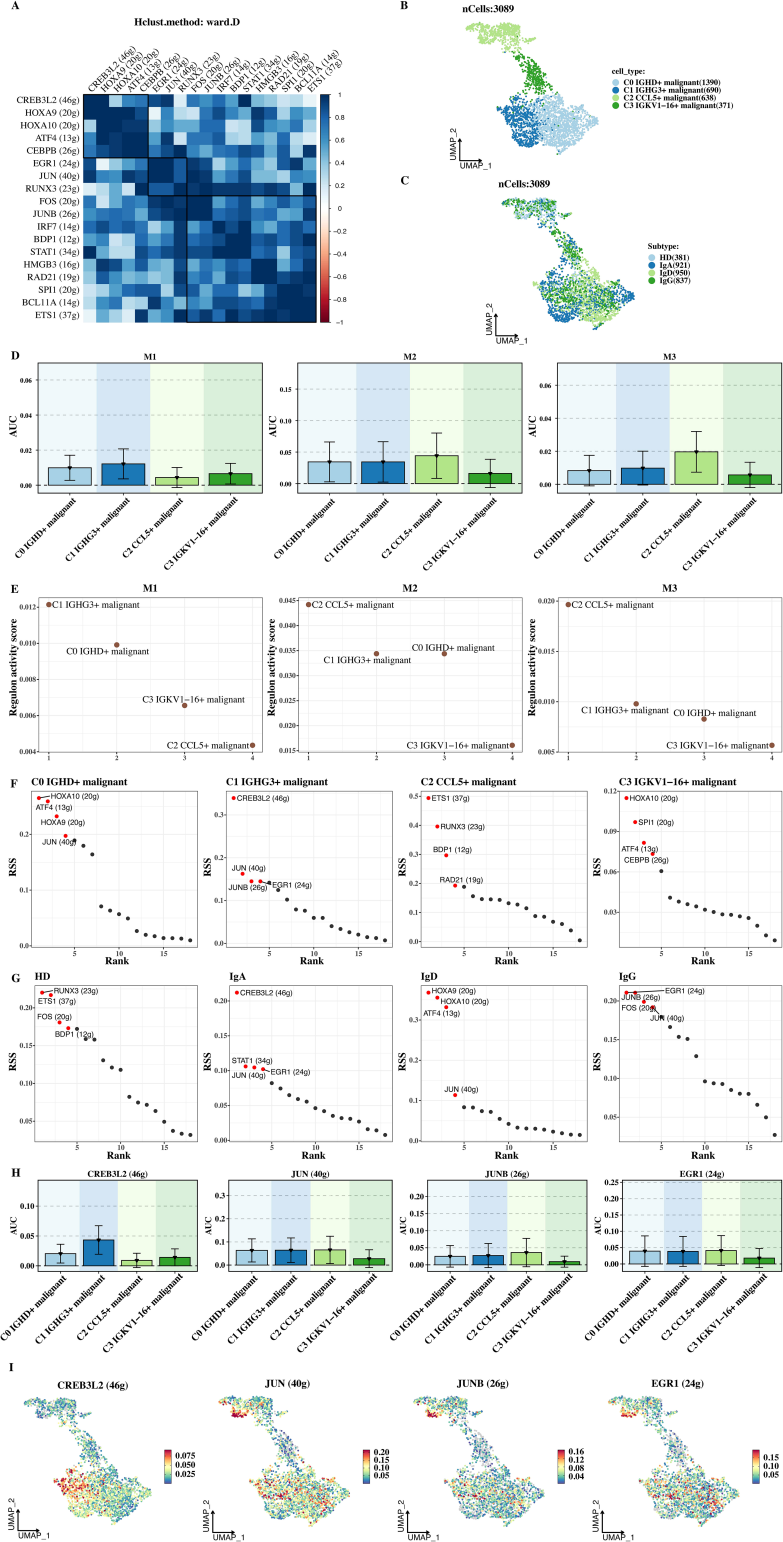
Transcription factor analysis. **(A)** Clustering of transcription factors into three groups (M1, M2, M3) based on the CSI matrix. **(B)** UMAP plot showing distribution of tumor cell subsets across transcription factor groups. **(C)** UMAP plot of tumor subtypes across transcription factor groups. **(D)** AUC values of transcription factors in each module, reflecting their regulatory activity. **(E)** AUC values of transcription factors in tumor subtypes. **(F)** RSS scores of transcription factors across tumor cell subtypes. **(G)** RSS scores of transcription factors in tumor subtypes. **(H)** AUC visualization of key transcription factors (e.g., CREB3L2) in C1 malignant cells. **(I)** AUC visualization of CREB3L2 in C1 malignant cells, highlighting its regulatory role.

After calculating the CSI matrix of each transcription factor, a clustering algorithm was used to cluster the transcription factors into 3 groups: M1, M2, and M3 (**Figure 5A**). Clustering analysis can classify transcription factors into different categories according to their similarities, facilitating the study of the functions and regulatory mechanisms of different transcription factor groups. The distribution of tumor cell subsets and different tumor subtypes was shown (**Figures 5B, C**). It was found that there are differences in the distribution of tumor cell subsets and tumor subtypes among different transcription factor groups.

The AUC values of transcription factors in each module were shown (**Figures 5D, E**). AUC is used to quantitatively measure the regulatory activity of transcription factors. The results showed that C1 malignant cells had a higher AUC in M1, indicating that the transcription factors in group M1 had a higher regulatory activity in C1 malignant cells and may play an important role in maintaining and developing the malignant phenotype of C1 cells. The RSS scores of transcription factors in each tumor cell subtype and tumor subtype were calculated and visualized (**Figures 5F, G**). The RSS score can express the specific regulatory degree of transcription factors in different cell subtypes. Through visualization, it can be seen that the RSS scores of different transcription factors in different tumor cell subtypes are different, further confirming the cell - subtype specificity of transcription factor regulation.

Finally, 4 transcription factors with advantages in C1 malignant cells (such as CREB3L2) were selected for AUC visualization (**Figures 5H, I**). Based on AUC visualization, the differences in the transcriptional regulatory activities of these 4 transcription factors in each sample cell group were intuitively shown. The AUC of CREB3L2 in C1 malignant cells was significantly higher than that in other cell types, indicating that it plays an important role in the transcriptional regulation of C1 malignant cells and providing an important target for further studying the molecular regulatory mechanism of C1 malignant cells.

### 3.7 Exploring the association between CREB3L2 and angiogenesis

**Figure 6:**
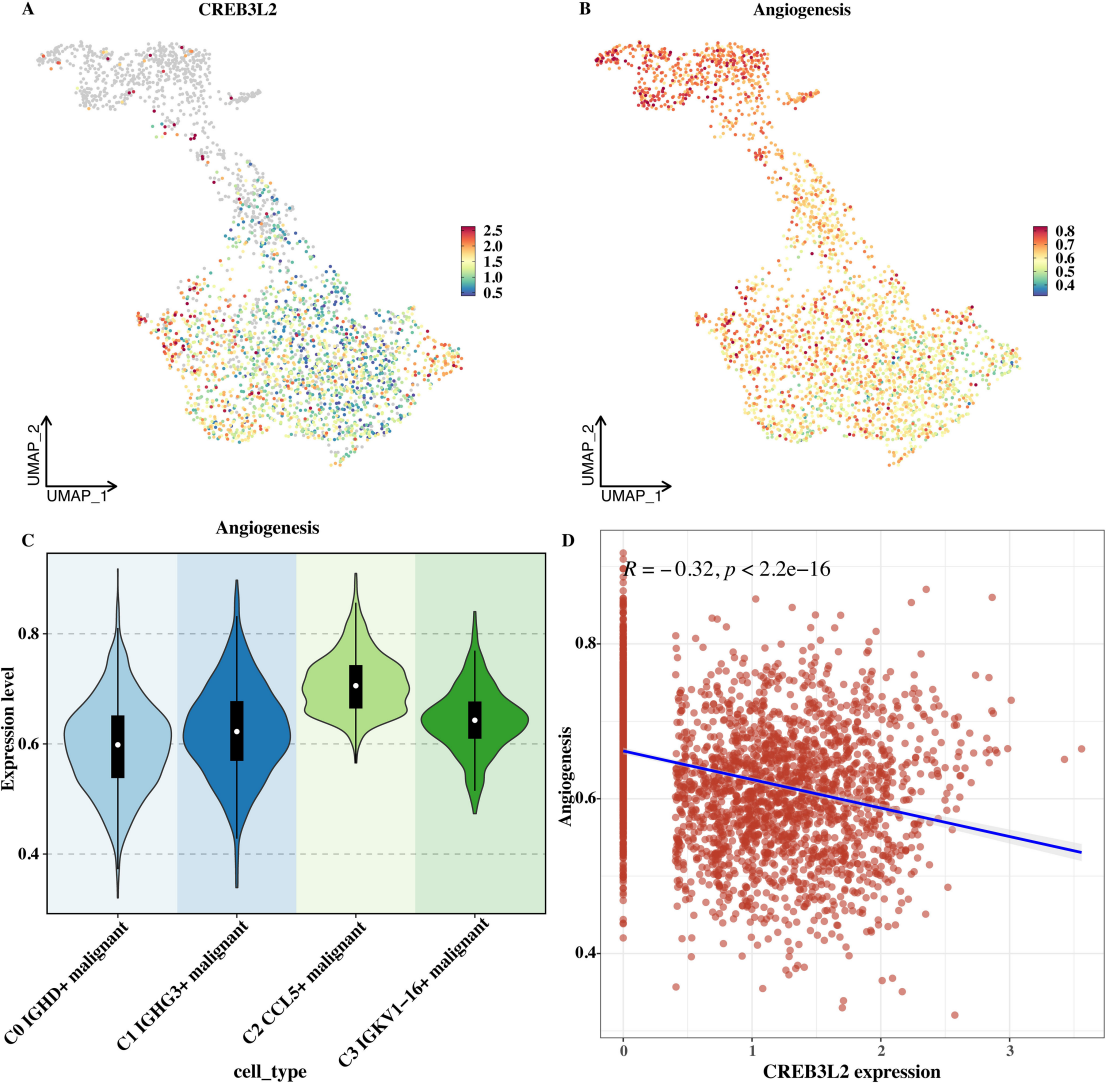
CREB3L2 expression and angiogenesis score in MM single cells. **(A)** UMAP plot of CREB3L2 expression in single cells. **(B)** UMAP plot of angiogenesis scores in single cells. **(C)** Violin plot of angiogenesis scores across malignant subtypes (C0–C3). Boxes represent median and interquartile range; whiskers indicate min–max. **(D)** Scatter plot showing a negative correlation between CREB3L2 log2 (TPM + 1) and angiogenesis score; Pearson’s r = -0.32, *P* < 0.001.

The ssGSEA algorithm was used to calculate the angiogenesis score in single - cell data, and the distributions of CREB3L2 and the angiogenesis score on the UMAP plot were shown respectively (**Figures 6A, B**). From the UMAP plot, the distribution patterns of CREB3L2 and the angiogenesis score at the single - cell level can be directly observed, and there are certain differences in their distributions. The distribution of the angiogenesis score in each malignant subtype cell was shown (**Figure 6C**). C2 and C3 (at the end of the differentiation trajectory) had high scores, while C0 and C1 had low scores, suggesting that the angiogenesis activity of tumor cells may gradually increase with differentiation.

A correlation analysis was performed with the expression level of CREB3L2, and the result showed a significant negative correlation (**Figure 6D**). This suggests that CREB3L2 may inhibit angiogenesis. Changes in its level may affect the angiogenesis ability of tumor cells and indirectly affect tumor growth and metastasis, providing a clue for a deeper understanding of the regulatory mechanism of tumor angiogenesis.

### 3.8 Role of CREB3L2 in proliferation, apoptosis, and migration of myeloma cells and its mechanisms

**Figure 7:**
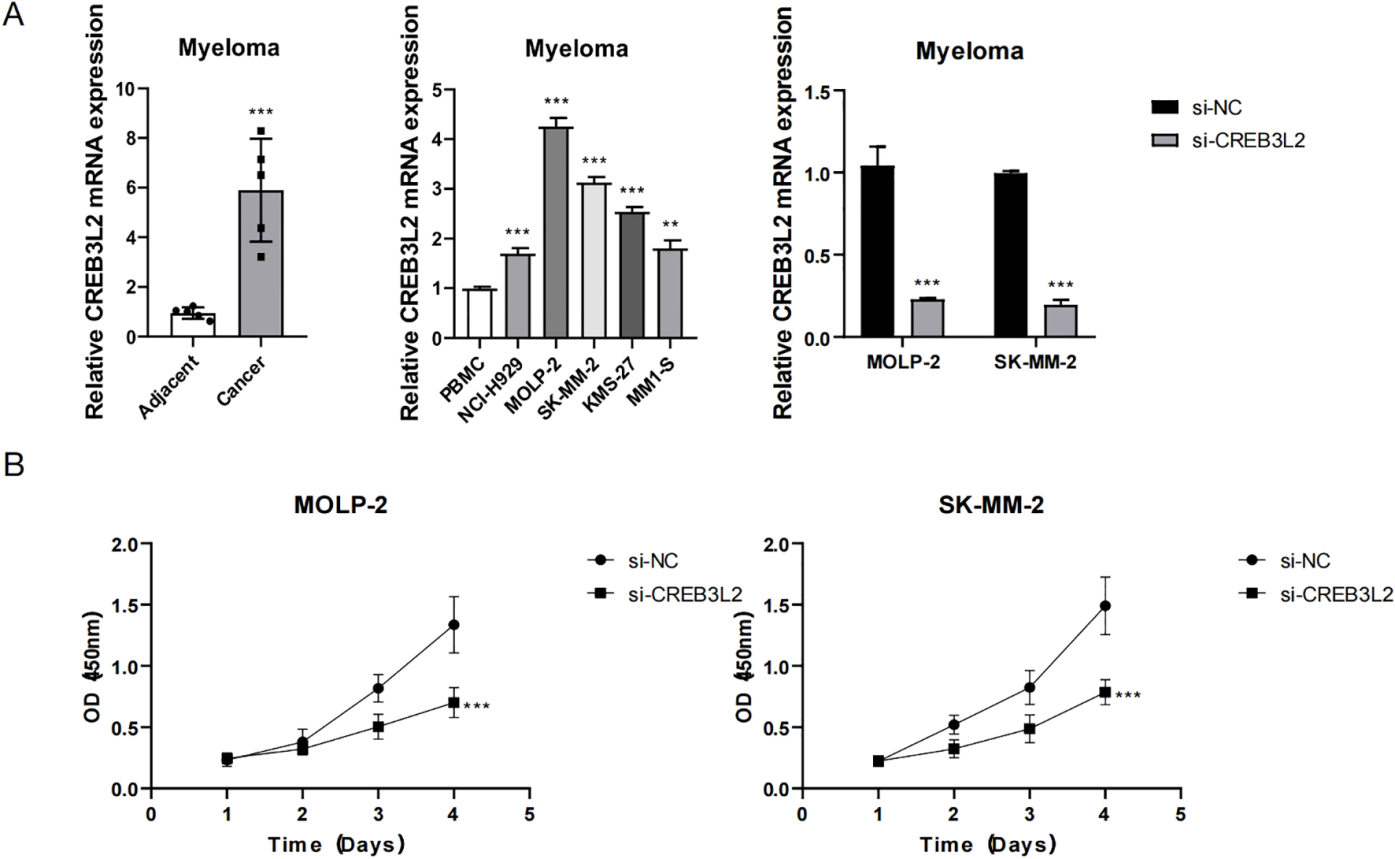
Role of CREB3L2 in myeloma cell proliferation. **(A)** Bar graphs showing relative CREB3L2 mRNA expression in paired MM cancer tissue versus adjacent normal bone marrow (left), myeloma cell lines (MOLP‐2, SK‐MM‐2) versus normal PBMCs (middle), and si‐CREB3L2 versus si‐NC groups (right). Data are mean ± SD from three independent experiments. **P < 0.01, ***P < 0.001 (Student’s two‐tailed t‐test). **(B)** Cell proliferation curves (CCK‐8 assay; OD450) in MOLP‐2 and SK‐MM‐2 cells transfected with si‐CREB3L2 or si‐NC over 1, 2, 3, and 4 day. Data are mean ± SD (n = 4). Two‐way ANOVA with Bonferroni’s *post hoc*: ***P < 0.001 for si‐CREB3L2 versus si‐NC at each time point.

**Figure 8:**
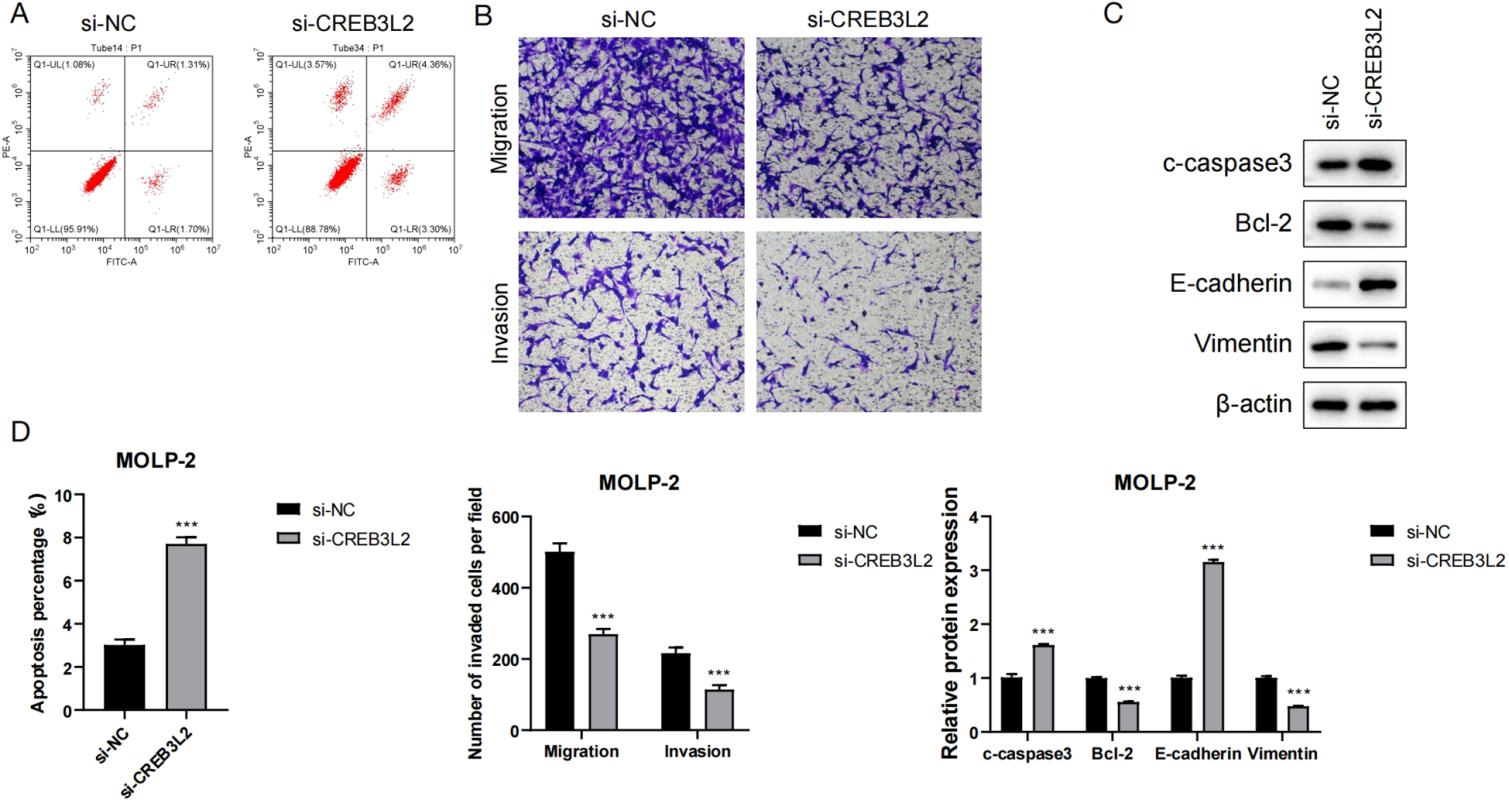
Role of CREB3L2 in apoptosis, migration, and EMT of myeloma cells. **(A)** Representative flow cytometry histograms and quantification of apoptotic rates in MOLP‐2 and SK‐MM‐2 cells of si‐CREB3L2 or si‐NC transfection. Data are mean ± SD (n = 3). ***P < 0.001 (Student’s two‐tailed t‐test). **(B)** Transwell migration and invasion assays for MOLP‐2 and SK‐MM‐2 cells. Left: representative images (×200); right: quantification of migrated/invaded cells per field. Data are mean ± SD (n = 3). **P < 0.01, ***P < 0.001 (Student’s two‐tailed t‐test). **(C)** Western blot showing expression of pro‐apoptotic (cleaved caspase‐3) and anti‐apoptotic (BCL‐2) proteins plus EMT markers (E‐cadherin, vimentin) in si‐CREB3L2 and si‐NC groups. **(D)** Quantitative analysis comparing apoptosis rates, migration/invasion cell numbers, and protein expression between groups. Data are mean ± SD (n = 3). **P < 0.01, ***P < 0.001 versus si‐NC (Student’s two‐tailed t‐test).

Further functional experiments revealed that CREB3L2 is not only associated with angiogenesis, but also directly affects the proliferation, apoptosis, and migration of myeloma cells, as shown in **Figures 7**, **8**. **Figure 7A** shows the expression characteristics of CREB3L2 gene in myeloma through three sets of histograms: the left histogram shows that the expression of CREB3L2 mRNA in the cancer tissue is significantly increased compared with the adjacent normal tissue (*P* < 0.001); the middle histogram detects different cell lines, showing that the expression of CREB3L2 in myeloma cell lines (MOLP-2, SK-MM-2) is significantly higher than that in normal PBMC cells (majority group, *P* < 0.001); the right histogram verifies that the gene expression of the si-CREB3L2 interference group is significantly down-regulated compared with that of the control group (si-NC) (*P* < 0.001), indicating that the interference efficiency is effective. **Figure 7B** shows the line graph of cell proliferation experiment. The results show that in MOLP-2 and SK-MM-2 cells, the OD value (450 nm) of the si-CREB3L2 group increased significantly lower than that of the si-NC group with time (*P* < 0.001), suggesting that the expression level of CREB3L2 is positively correlated with the proliferation ability of myeloma cells. Knocking down this gene can significantly inhibit cell proliferation.

**Figure 8A** shows that the percentage of apoptotic cells in the si-CREB3L2 group was significantly higher than that in the si-NC group (*P* < 0.001), indicating that knockdown of CREB3L2 can induce apoptosis of myeloma cells. **Figure 8B** By Transwell experiments, it was found that the number of migrating and invading cells in the si-CREB3L2 group was significantly lower than that in the control group, suggesting that knockdown of this gene can inhibit the motility of cells. The Western blot results in **Figure 8C** showed that the expression of pro-apoptotic protein c-caspase3 and epithelial marker protein E-cadherin was up-regulated in the si-CREB3L2 group, while the expression of anti-apoptotic protein Bcl-2 and interstitial marker protein Vimentin was down-regulated, suggesting that CREB3L2 may be involved in the regulation of epithelial-mesenchymal transition (EMT). The quantitative analysis of **Figure 8D** further verified the above results, and the differences in apoptosis rate, number of migrating/invading cells, and protein expression between groups reached extremely significant levels (*P* < 0.001). In summary, knocking down CREB3L2 can play an anti-tumor role by promoting apoptosis, inhibiting EMT, and cell migration and invasion.

Previous studies indicate that in triple-negative breast cancer, CREB3L2 cleavage produces an active fragment that promotes Hedgehog signaling via GLI1, which not only enhances tumor proliferation and stress survival but also induces EMT by repressing E-cadherin and upregulating vimentin (38); CREB3L2 interacts with ATF6 to regulate EMT-TFs SNAI1/ZEB1 through CHOP and XBP1, correlating with decreased E-cadherin and increased vimentin in epithelial tumors (39); FUS-CREB3L2 fusions bind promoters of anti-apoptotic BCL2L1 and MCL1, and CREB3L2 knockdown increases Annexin V positivity, mirroring our apoptosis data (40); and CREB3L2 occupies promoters of ER-homeostasis genes (HSPA5, EIF2α) and apoptosis regulators (BAX, BAK), with its silencing upregulating pro-apoptotic transcripts and downregulating CCND1 and CDK4 (41). These findings support our observations that CREB3L2 knockdown in myeloma cells increases E-cadherin, decreases vimentin, upregulates Bax, and downregulates Bcl-2, indicating suppression of EMT-like and anti-apoptotic programs. Future RNA-seq and ChIP-seq analyses will confirm these direct targets.

### 3.9 Construction of a prognostic model with the CREB3L2 regulatory network and angiogenesis genes

**Figure 9:**
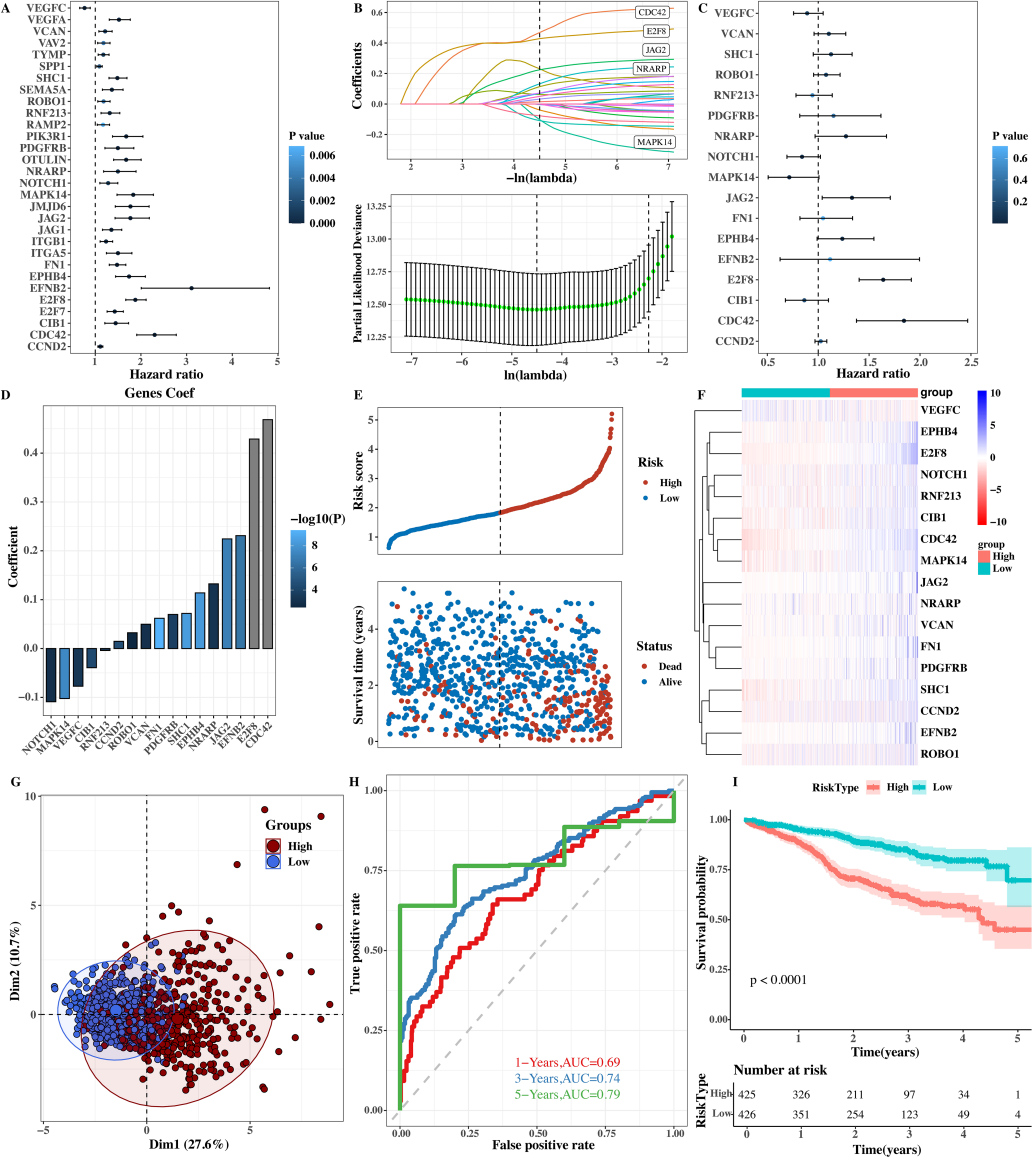
Prognostic model based on CREB3L2 and angiogenesis genes. **(A)** Forest plot of univariate Cox regression analysis for 75 candidate genes. P values and hazard ratios (HR) with 95% confidence intervals (CI) are shown. **(B)** LASSO‐Cox model construction: coefficient profiles and cross‐validation (10‐fold) error curves. **(C)** Forest plot of multivariate Cox regression for final prognostic signature (n = 6 genes). HR (95% CI) and P values are indicated. **(D)** Coefficient values for each gene in the risk score formula. **(E)** Scatter plot of risk score versus overall survival time; correlation assessed. **(F)** Heatmap of expression levels for prognostic genes in low‐ versus high‐risk groups. **(G)** PCA plot demonstrating separation of low‐ and high‐risk groups (PC1 = 27.6%, PC2 = 10.7%). **(H)** Time‐dependent ROC curves: AUC = 0.69 (1 year), 0.74 (3 years), 0.79 (5 years). **(I)** Kaplan–Meier survival curves: median cutoff stratifies patients into low‐ and high‐risk. Log‐rank P < 0.0001.

A total of 75 genes, including TARGET genes (importance > 10) related to the specific transcription factor CREB3L2 of C1 tumor cells and angiogenesis genes, were used for univariate Cox analysis to screen out genes related to survival (**Figure 9A**). Univariate Cox analysis preliminarily judged the relationship between these genes and the survival outcome of patients, providing important candidate genes for the next - step construction of the prognostic model.

A prognostic model was successfully constructed using the LASSO + Cox algorithm (**Figure 9B**). The LASSO algorithm compresses and selects regression coefficients during model construction to avoid overfitting and selects the gene combination with the most predictive value for prognosis. The forest plot of each prognostic gene was shown by multivariate COX analysis (**Figure 9C**). In the forest plot, the hazard ratio and confidence interval of each prognostic gene are clearly shown, which can directly reflect the degree of risk impact of each gene on the survival of patients. The model coef values of each gene were shown (**Figure 9D**). The coef value indicates the weight of each gene in the model, and its magnitude and positive/negative sign reflect the direction and degree of contribution to the risk score.

Scatter plots of risk scores and survival times (**Figure 9E**) and heat maps of the expression levels of prognostic genes (**Figure 9F**) were given. From the scatter plot, the relationship between the risk score and survival time can be seen. It can be found that patients with higher risk scores have shorter survival times. The heat map shows the expression of prognostic genes in different samples. Different colors represent high and low gene expression levels, and the expression differences of prognostic genes among different patients can be seen through the heat map. The principal component analysis (PCA) results of reducing the dimensionality of prognostic gene expression levels (**Figure 9G**) were shown. PCA analysis can reduce the dimensionality of high - dimensional gene expression data and show the distribution of samples in low - dimensional space. Differences between samples in different risk groups can also be seen from PCAT.

The high - and low - risk groups were stratified by the median value, and further survival analysis (**Figure 9H**) and timeROC results (**Figure 9I**) were shown. From the survival analysis diagram, the survival rate of high - risk group patients was lower than that of low - risk group patients, and the difference was statistically significant (*P* < 0.0001). From the timeROC diagram, it can be seen that the AUC values of this model at 1 year, 3 years, and 5 years were 0.69, 0.74, and 0.79 respectively. This model has good prediction performance and can effectively predict the prognosis of MM patients.

### 3.10 Functional analysis of High - and Low - risk groups

**Figure 10:**
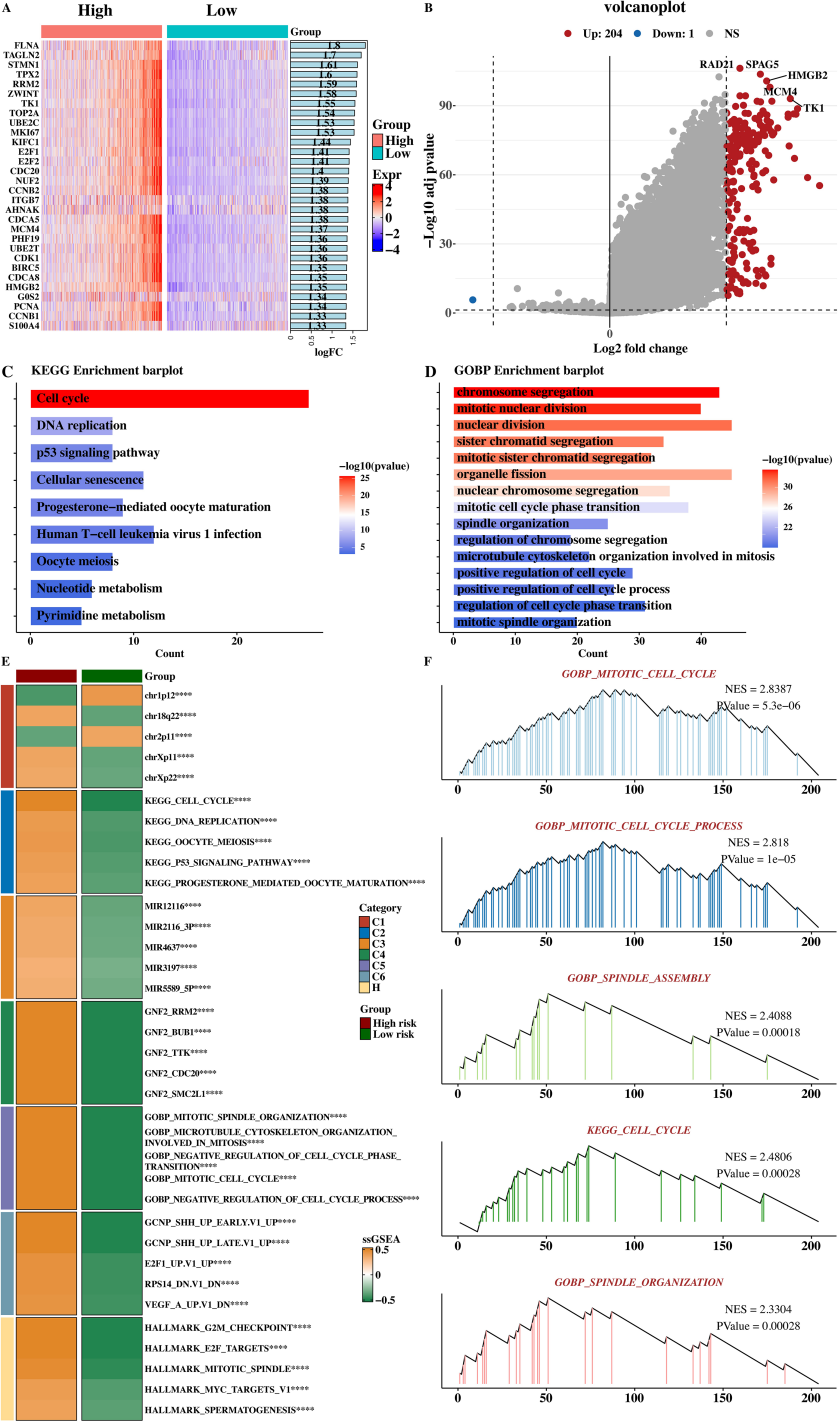
Functional analysis of high- and low-risk groups. **(A)** Heatmap of the top 30 differentially expressed genes between high‐risk and low‐risk patients (|log2 FC| > 1, adj *P* < 0.05). **(B)** Volcano plot: red dots, upregulated; blue dots, downregulated (adj *P* < 0.05). **(C)** KEGG enrichment analysis of upregulated genes in high‐risk group; dot size reflects gene count, color indicates –log10 (adj P). **(D)** GOBP enrichment showing significantly overrepresented biological processes (adj *P* < 0.05). **(E)** Heatmap of ssGSEA pathway scores (|NES| > 1, adj *P* < 0.05). **(F)** GSEA plots for hallmark pathways (G2/M checkpoint, E2F targets); nominal *P* < 0.01, FDR < 0.05. ****p < 0.0001.

The limma software (v3.48.3; Australia) was used to analyze the differential genes between the high - and low - risk groups. The top 30 genes ranked by logFC were selected, and the expression heat map of differential genes was shown (**Figure 10A**). Different colors in the heat map represent different gene expression levels, and the pattern of gene expression differences between the high - and low - risk groups can be intuitively observed through the heat map. The results of differential genes were visualized by a volcano plot (**Figure 10B**). In the volcano plot, red dots represent up - regulated genes, blue dots represent down - regulated genes, and the size and color depth of the dots represent the significance of gene expression differences, clearly showing the distribution of differentially expressed genes between the high - and low - risk groups.

KEGG and GOBP enrichment analyses were performed on the up - regulated genes in the high - risk group, and the KEGG enrichment result (**Figure 10C**) and GOBP enrichment result (**Figure 10D**) were shown respectively. KEGG enrichment analysis showed that the up - regulated genes in the high - risk group were highly enriched in tumor - related pathways such as the cell cycle, DNA replication, and PI3K - Akt signaling pathway. The GOBP enrichment analysis result showed that these genes were highly enriched in biological processes such as mitotic cell cycle progression and spindle assembly, suggesting that the tumor cells of high - risk group patients have higher proliferative ability and abnormal cell cycle progression.

The scores of functional pathways in each msigdb were counted, and the pathways with significant differences between the high - and low - risk groups were selected to draw a heat map (**Figure 10E**). The heat map shows the activity differences of these pathways between the high - and low - risk groups, verifying the biological function differences between the high - and low - risk groups again. Five GSEA results of up - regulated genes in the high - risk group were presented (**Figure 10F**), showing that these genes were significantly related to the cell cycle and mitosis, such as enrichment in pathways like the G2M checkpoint and E2F targets. This is consistent with the KEGG and GOBP enrichment results and further explores the biological characteristics and malignant progression mechanisms of high - risk group tumor cells.

### 3.11 Immune infiltration, mutation, tumor immune dysfunction and exclusion, and drug sensitivity analysis

**Figure 11:**
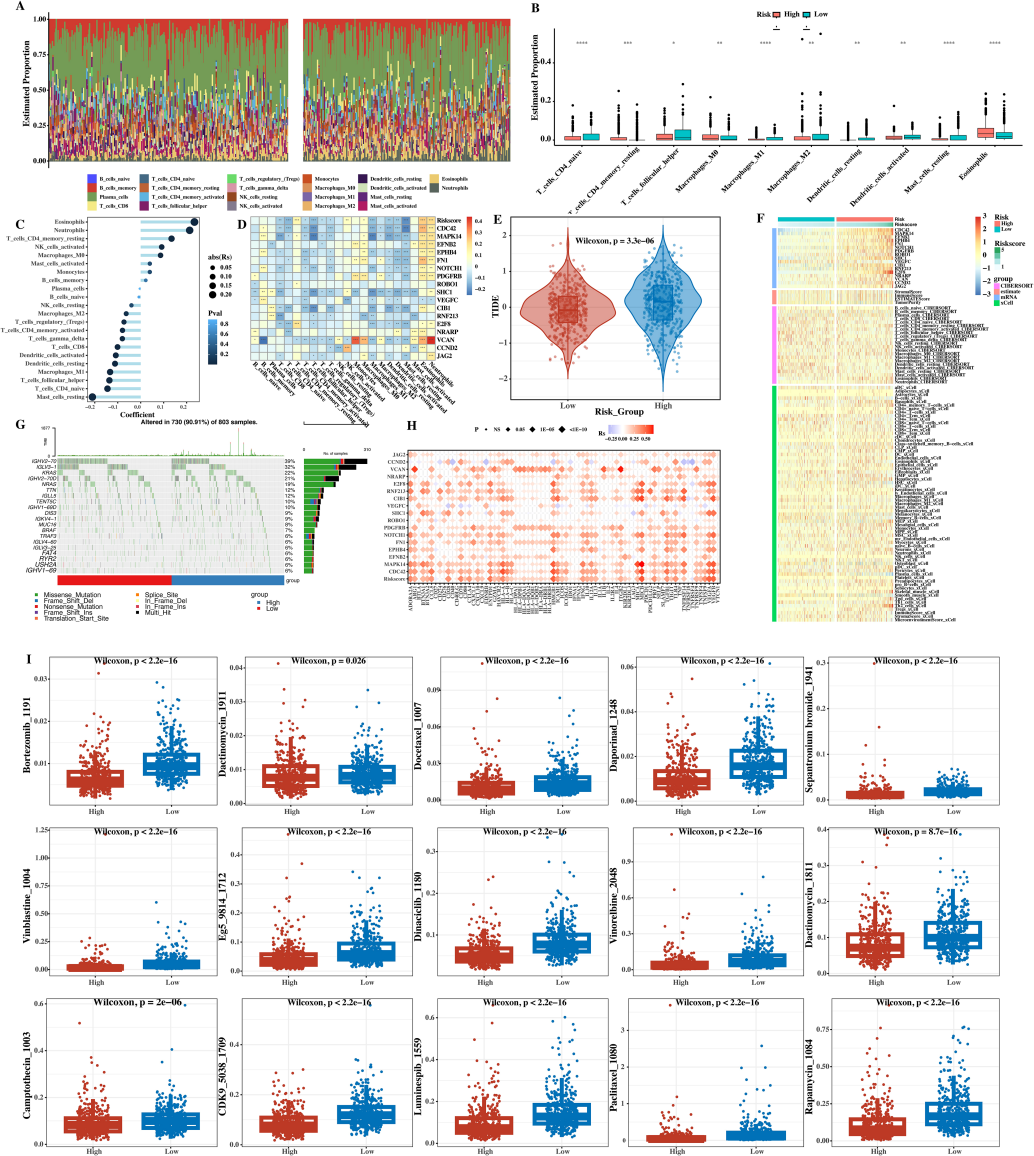
Immune infiltration, mutation, TIDE, and drug sensitivity analysis. **(A)** Bar chart of relative proportions of 22 immune cell types (CIBERSORT) in high‐ versus low‐risk groups. **(B)** Box plot comparing proportions of selected immune cells; *P < 0.05, **P < 0.01, ***P < 0.001 (Wilcoxon rank‐sum test). **(C)** Bar graph of Pearson’s correlation between risk score and immune cell fractions: r and P values are indicated for each cell type. **(D)** Heatmap of correlations between immune cells and prognostic gene expression (Pearson’s r; *P < 0.05, **P < 0.01, ***P < 0.001). **(E)** TIDE scores in low‐ versus high‐risk groups; ***P < 0.001 (Student’s two‐tailed t‐test). **(F)** Heatmap showing combined prognostic gene expression and immune/stromal scores (ESTIMATE, CIBERSORT, xCell). **(G)** Waterfall plots of top 20 somatic mutations in high‐ and low‐risk groups. **(H)** Bubble plot of Pearson’s correlation between immune checkpoint genes, prognostic genes, and risk scores. **(I)** Box plot of predicted drug IC50 for 15 agents. ****p < 0.0001.

Bar charts (**Figure 11A**) and box plots (**Figure 11B**) of the proportions of immune cells predicted by CIBERSORT in the two groups were made. Through these visual charts, the changes in the proportions of immune cells in the high - and low - risk groups can be seen. A bar - graph of the correlation analysis between the immune cells predicted by CIBERSORT and the risk score was drawn (**Figure 11C**). Through the bar - graph, it was found that the proportions of some immune cells were significantly correlated with the risk score, further verifying the important position of immune cells in the prognosis of MM.

A heat map of the correlation between immune cells and prognostic genes was drawn (**Figure 11D**). The color depth in the figure represents the strength of the correlation. From the heat map, the complex relationships between different immune cells and prognostic genes can be seen, indicating that immune cells may affect the prognosis of patients by acting on the expression of prognostic genes. The TIDE was used to calculate the immunotherapy situation, and it was found that there were significant differences in TIDE scores between the two groups (**Figure 11E**). The TIDE score can be used to evaluate the responsiveness of tumors to immunotherapy. The high - risk group had a higher TIDE score, indicating that it may respond poorly to immunotherapy, providing a reference for the selection of immunotherapy regimens in clinical practice.

A heat map showing the expression levels of prognostic genes and the immune infiltration levels predicted by Estimation of Stromal and Immune cells in Malignant Tumor tissues using Expression data (ESTIMATE), Cell type Identification By Estimating Relative Subsets Of RNA Transcripts (CIBERSORT), and cell type enrichment analysis (xCell) was presented (**Figure 11F**), integrating multiple datasets to fully display the correlation between prognostic gene expression and immune infiltration levels and helping to gain a deeper understanding of the relationship between the tumor microenvironment and prognosis. The top 20 gene mutation waterfall plots of the high - and low - risk groups were shown (**Figure 11G**), showing the mutation types, sites, etc. of each mutated gene. It was found that the gene mutation spectra of the high - and low - risk groups were different, and the mutations of some genes were related to the poor prognosis of the high - risk group.

The correlations between immune checkpoint genes, prognostic genes, and risk scores were calculated and presented using a bubble plot (**Figure 11H**). The size and color of the bubbles in the bubble plot represent the strength of the correlation. From the figure, it can be seen that some immune checkpoint genes have certain correlations with prognostic genes and risk scores, providing potential directions for the selection of immunotherapy targets. Finally, the differences in the sensitivity of 15 drugs between the two groups of patients were shown (**Figure 11I**). The box plot shows the sensitivity distribution of different drugs in the high - and low - risk groups. It was found that high - risk group patients had lower sensitivity to some drugs (such as some chemotherapy drugs), while they may have differential sensitivity to other drugs (such as targeted drugs), providing a reference for personalized drug use in clinical practice for different patients.

## 4 Discussion

We systematically analyzed bulk transcriptome and scRNA‐seq data from MM patients to elucidate how tumor‐cell heterogeneity and angiogenesis‐related genes influence prognosis. By subsetting malignant cells, we identified distinct tumor‐cell subpopulations. InferCNV revealed extensive genomic copy‐number alterations, with CNV scores differing significantly among subgroups—reflecting variation in malignancy and developmental state. Pseudotime analysis reconstructed differentiation trajectories, pinpointing dynamic transitions of tumor‐cell clusters. SCENIC‐based TF analysis uncovered TF regulatory‐network differences across subpopulations, highlighting core regulators in specific clusters.

In the research on the correlation between angiogenesis genes and prognosis, it was found that angiogenesis genes are involved in the pathogenesis and progression of MM. The prognostic model based on angiogenesis genes and transcription factors can effectively predict the prognosis of patients. The model integrates multiple gene information and has a good prognostic effect, providing data reference for clinical treatment. In addition, through the functional analysis of high - and low - risk groups, it was found that high - risk groups had high expression of related genes, which were related to biological processes such as the cell cycle and mitosis. That is, the tumor cells of high - risk group patients have high proliferative ability and malignancy, which is the reason for the poor prognosis of high - risk group patients.

This study found that through immune infiltration, mutation, TIDE, and drug sensitivity analyses, the characteristics of the immune microenvironment, gene mutation status, and differences in sensitivity to immunotherapy and drug therapy of MM patients were obtained. From the immune infiltration analysis, significant differences were found in the composition and proportion of immune cell types between high - and low - risk groups. There are complex correlations between immune cells, prognostic genes, and risk scores, indicating that the immune microenvironment plays an important role in the occurrence, development, and prognosis of MM. The increase in immunosuppressive cells in the high - risk group may inhibit the body’s anti - tumor immune response and promote tumor development, while the relatively large number of immune - activating cells in the low - risk group is conducive to the body’s immune surveillance and clearance of tumors. The results of gene mutation analysis suggest that high - and low - risk groups have different gene mutation spectra. Some gene mutations may affect the biological behavior changes (proliferation, apoptosis, drug resistance) of tumor cells, thus affecting the prognosis of patients.

The results of TIDE analysis suggest that there are significant differences in the immunotherapy responsiveness between high - and low - risk groups. Since a higher TIDE score indicates a high enrichment of immunosuppressive cells and high expression of immune checkpoint molecules in the microenvironment, the high - risk group has a poor response to immunotherapy. In contrast, the low - risk group may have a better response to immunotherapy. This has great application value in the selection of clinical immunotherapy regimens and can achieve the goal of precise immunotherapy for MM patients.

Drug sensitivity analysis shows that there are obvious differences in the sensitivity of 15 drugs between the two groups of patients. High - risk group patients have low sensitivity to some chemotherapy drugs, which is related to the drug - resistance mechanism of tumor cells. For targeted drugs, the sensitivities between risk groups vary, providing a basis for personalized drug treatment in clinical practice. The study of drug sensitivity can analyze the risk grouping of patients and their gene characteristics, and based on the evidence of potential drug effectiveness, more targeted drugs can be given to patients, improving the treatment effect and avoiding unnecessary therapeutic side - effects.

The prognostic model presented in this study was developed and internally validated using cohorts derived from the UCSC Xena and GEO databases, but it has not yet been evaluated on an independent, external dataset. As a consequence, its robustness and generalizability remain uncertain when applied to patient populations beyond those originally analyzed. Without external validation, there is a risk of overfitting to specific cohort characteristics (e.g., demographic or technical batch effects) that may not generalize to other clinical settings. Therefore, caution is warranted before implementing this model in routine clinical practice: prospective validation in geographically and ethnically diverse cohorts—as well as across multiple sequencing platforms—is necessary to confirm its predictive accuracy and stability. In addition, the lack of external benchmarking may limit the model’s ability to account for heterogeneity in sample collection procedures, treatment regimens, and follow-up durations that are common in real-world settings. Future studies should prioritize obtaining independent validation datasets—ideally from multi-center clinical trials or publicly available consortia such as MMRF CoMMpass or other international miRNA/RNA-seq repositories—to assess the model’s performance under diverse conditions. Only with such external validation can the true clinical translation potential of our angiogenesis- and transcription factor-based prognostic signature be fully established.

Compared with earlier reports on tumor-associated endothelial heterogeneity—Zhao et al, who characterized endothelial cell diversity and plasticity in solid tumors at single-cell resolution (4)—our work extends these observations into the MM microenvironment by integrating single-cell pseudotime trajectory analysis with a comprehensive TF regulatory‐network framework. Our study (1) applies single-cell trajectory reconstruction to delineate the dynamic evolution of MM malignant subpopulations (C0–C3) with divergent angiogenic signatures; (2) directly link CREB3L2 TF activity to angiogenesis at the single-cell level by leveraging SCENIC‐inferred regulons; and (3) construct a prognostic signature that combines CREB3L2‐mediated transcriptional control with angiogenesis‐related gene expression. This dual‐layered approach not only reveals that high CREB3L2 activity in C1 cells correlates with suppressed angiogenic potential, but also demonstrates the utility of TF‐target network inferences for pinpointing drivers of MM vascular remodeling. Thus, while prior studies have described endothelial heterogeneity in the tumor milieu, our work uniquely (i) captures the trajectory‐dependent orchestration of TF‐driven angiogenic programs in MM tumor cells, (ii) provides experimental validation that CREB3L2 knockdown modulates both angiogenesis and EMT‐related targets (e.g., increased E-cadherin, reduced vimentin), and (iii) delivers a prognostic model rooted in both single‐cell regulatory logic and bulk transcriptomic data. Consequently, these innovations establish a novel conceptual framework for understanding how transcriptional regulators such as CREB3L2 dynamically repress angiogenesis during myeloma progression—insights that were not addressed by existing single-cell endothelial atlases.

Despite leveraging large-scale bulk transcriptomic and single-cell RNA-sequencing data, our study has several important limitations. First, although we analyzed 851 bulk MM samples from UCSC Xena and performed single-cell profiling on 4 MM tumor samples (with 5 healthy controls), this sample size remains modest given the well-known heterogeneity of multiple myeloma. Consequently, our findings may not be fully generalizable to all clinical subgroups, particularly rare cytogenetic or high-risk patients. Second, reliance on publicly available databases (e.g., UCSC Xena, GEO) potentially introduces selection biases. Variability in sample procurement, processing protocols, and clinical annotation across centers may affect data quality and compromise the uniformity of patient cohorts. Third, while we conducted extensive *in vitro* functional assays to validate the role of CREB3L2 in regulating angiogenesis, proliferation, apoptosis, and EMT markers, no *in vivo* experiments (e.g., xenograft or transgenic mouse models) were performed to confirm these mechanisms under physiological conditions. Finally, the retrospective nature of our analysis precludes assessment of longitudinal changes in CREB3L2 expression or angiogenesis signatures over the course of therapy. To address these limitations, future studies should incorporate larger, multi-center cohorts—preferably with prospective clinical sampling—and employ *in vivo* models to validate the prognostic model and mechanistic insights. Such efforts will be critical to confirm the translational relevance of CREB3L2-mediated pathways in MM and to refine personalized therapeutic strategies.

## 5 Conclusion

In this study, we performed an integrative analysis combining bulk transcriptomic data and single‐cell RNA sequencing to characterize tumor‐cell heterogeneity and investigate the prognostic significance of angiogenesis‐related genes in MM. We identified four distinct malignant cell subpopulations (C0–C3) with divergent copy number variation profiles, differentiation trajectories, and transcriptional regulatory patterns. Notably, high CREB3L2 activity in the C1 subpopulation was associated with suppressed angiogenic signaling, decreased proliferative and migratory capacity, and enhanced apoptotic propensity—findings corroborated by both in silico regulon inference and *in vitro* functional assays. By integrating CREB3L2‐target networks with a curated angiogenesis gene set, we constructed and internally validated a robust LASSO–Cox prognostic signature that accurately stratifies MM patients into high‐risk and low‐risk cohorts. Patients classified as high risk exhibited elevated expression of pro‐angiogenic and cell‐cycle‐related genes, increased immune‐suppressive cell infiltration, a higher tumor mutation burden, and reduced sensitivity to standard chemotherapeutics and immunotherapy. Conversely, the low‐risk group demonstrated a more favorable immune microenvironment and drug response profile. These results extend our understanding of MM biology by delineating the dynamic interplay between transcription factor–driven regulatory networks (particularly CREB3L2) and angiogenic programs at single‐cell resolution, highlighting the prognostic utility of combining single‐cell regulatory features with bulk gene‐expression signatures, and revealing potential therapeutic targets—for example, CREB3L2‐mediated pathways—that may be exploited to inhibit aberrant angiogenesis and overcome treatment resistance.

Despite the retrospective design, modest sample size, and absence of *in vivo* validation, our findings provide a solid theoretical foundation for future translational research. Prospective, multicenter studies involving larger, ethnically diverse MM cohorts are warranted to externally validate the prognostic model, and functional studies employing *in vivo* MM models (e.g., patient‐derived xenografts) should confirm the causal role of CREB3L2 and its downstream effectors in regulating angiogenesis, proliferation, and apoptosis. Moreover, given the observed correlations between angiogenic activity, immune‐suppressive cell infiltration, and drug sensitivity, follow‐on investigations should explore combination strategies that concurrently target CREB3L2‐driven transcriptional programs and immune checkpoint pathways in preclinical and clinical trial settings. In summary, our work elucidates critical mechanisms underlying MM progression, provides a novel prognostic signature grounded in both transcriptional regulation and angiogenesis, and identifies promising avenues for personalized therapeutic interventions aimed at improving outcomes for patients with MM.

## Data Availability

The original contributions presented in the study are included in the article/**Supplementary Material**. Further inquiries can be directed to the corresponding authors.
