# Supplementary material for: Investigating the role of tumor cell heterogeneity and angiogenesis genes in the prognosis of multiple myeloma
Source: Front Immunol. 2025 Jun 25;16:1610833. doi: 10.3389/fimmu.2025.1610833 (PMC12238041; doi:10.3389/fimmu.2025.1610833)
Supplement: Supplementary file 1 [file Table1.docx]

<https://www.jianguoyun.com/p/DZxsAHIQzc6eChjSwv4FIAA>
