# Supplementary material for: Investigating the role of tumor cell heterogeneity and angiogenesis genes in the prognosis of multiple myeloma
Source: Front Immunol. 2025 Jun 25;16:1610833. doi: 10.3389/fimmu.2025.1610833 (PMC12238041; doi:10.3389/fimmu.2025.1610833)
Supplement: Supplementary file 2 [file Table2.docx]

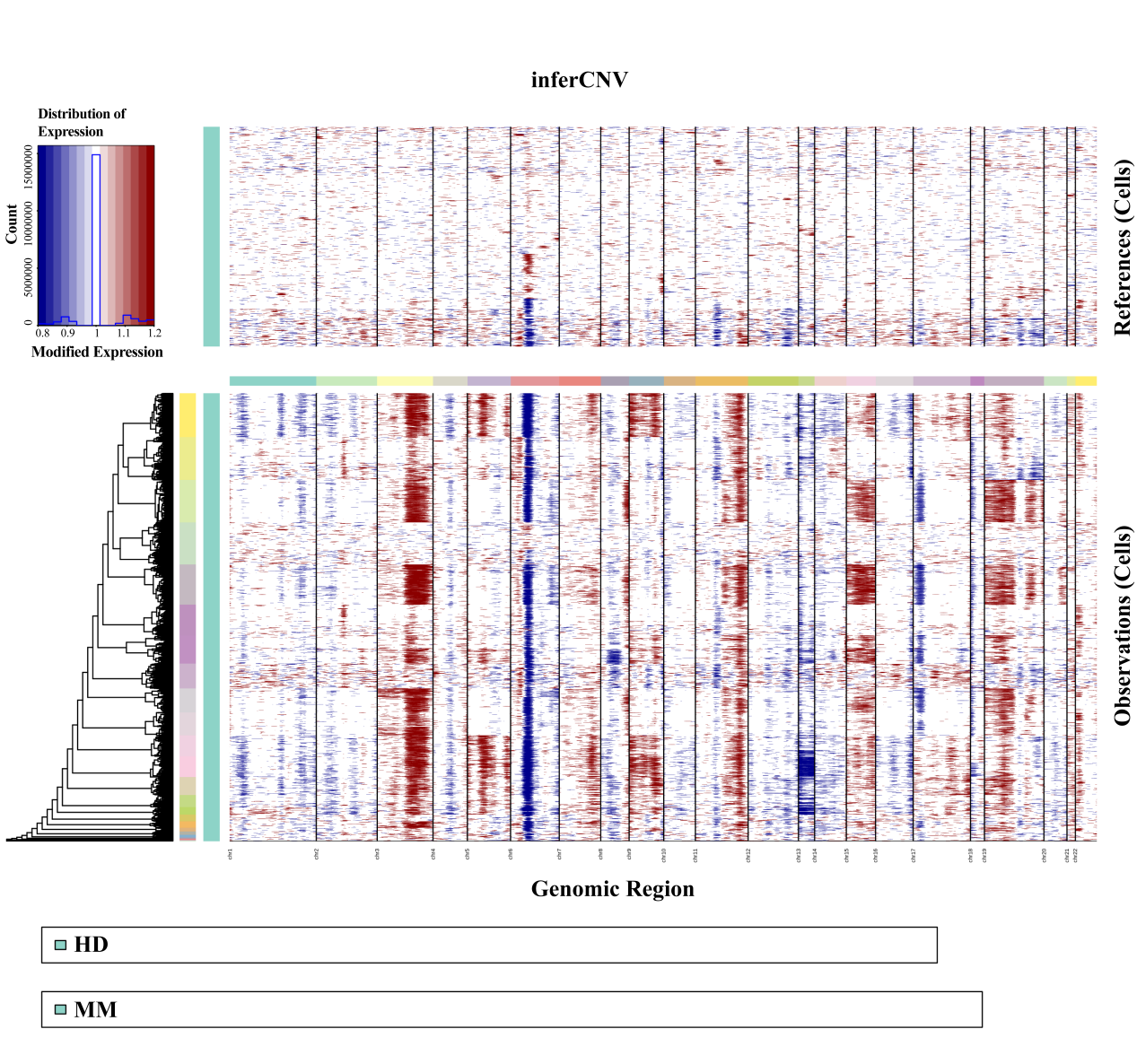


**Supplementary Figure 1: Heatmap of plasma cell analysis results using the inferCNV software, illustrating genomic copy number variation (CNV) across different samples.**


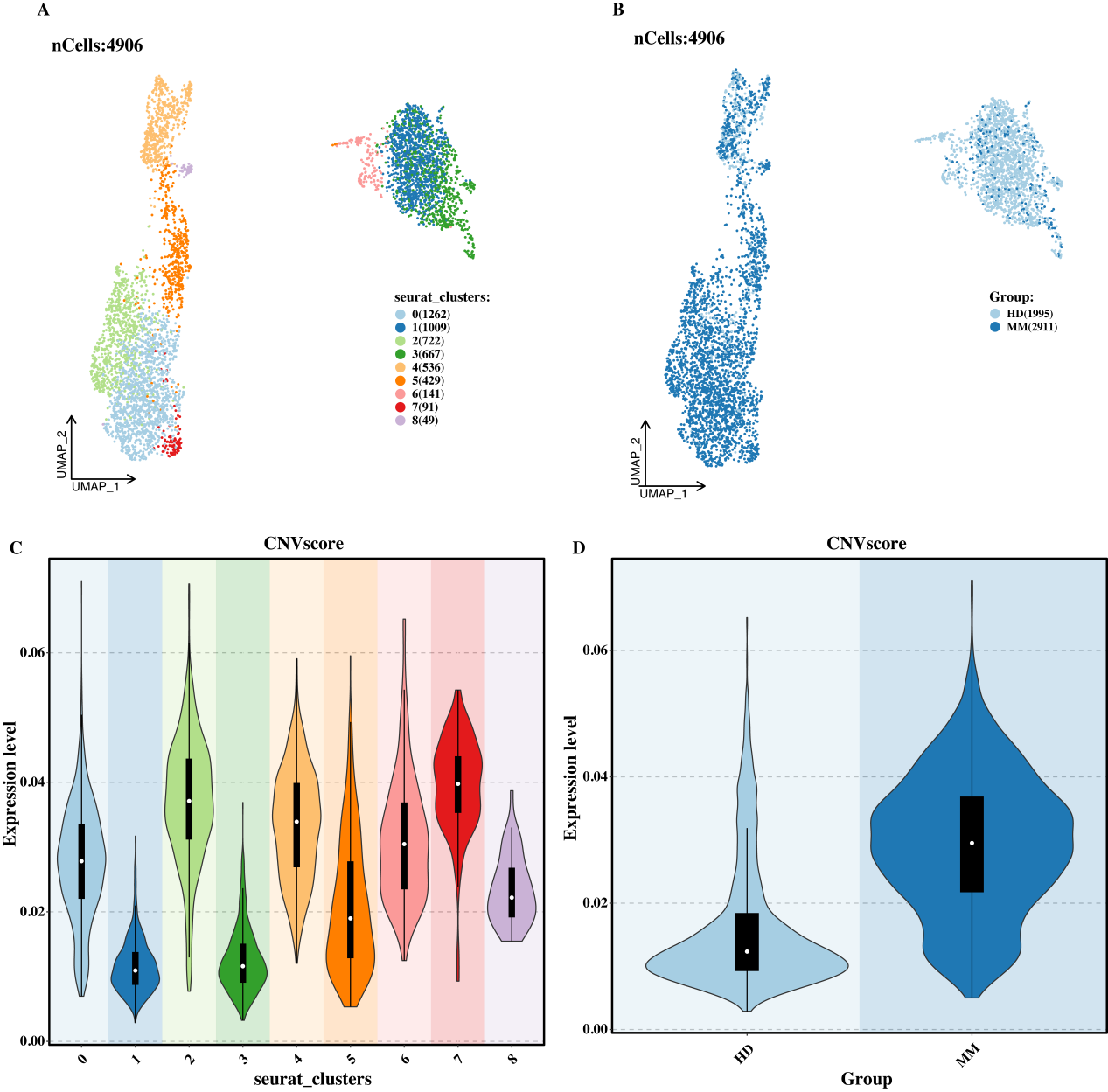


**Supplementary Figure 2: Analysis of plasma cell subsets.**

**(A)** UMAP plot of 4906 cells, colored by "seurat_cluster" (clusters 0 - 8). **(B)** UMAP plot showing MM (dark blue, 2911 cells) and HD (light blue, 1986 cells) samples. **(C)** Violin plot of CNVscore distribution across "seurat_cluster" subsets. **(D)** Violin plot showing CNVscore distribution in MM and HD samples.
